# Hydride‐Induced Reconstruction of Pd Electrode Surfaces: A Combined Computational and Experimental Study

**DOI:** 10.1002/adma.202410951

**Published:** 2024-12-04

**Authors:** Apinya Ngoipala, Christian Schott, Valentin Briega‐Martos, Minaam Qamar, Matous Mrovec, Sousa Javan Nikkhah, Thorsten O. Schmidt, Lewin Deville, Andrea Capogrosso, Lilian Moumaneix, Tanja Kallio, Arnaud Viola, Frédéric Maillard, Ralf Drautz, Aliaksandr S. Bandarenka, Serhiy Cherevko, Matthias Vandichel, Elena L. Gubanova

**Affiliations:** ^1^ School of Chemical Sciences and Chemical Engineering Bernal Institute University of Limerick Limerick V94 T9PX Ireland; ^2^ Physics of Energy Conversion and Storage Department of Physics Technical University of Munich James‐Franck‐Straße 1 85748 Garching Germany; ^3^ Helmholtz Institute Erlangen‐Nürnberg for Renewable Energy (IET‐2) Forschungszentrum Jülich GmbH Cauerstr. 1 91058 Erlangen Germany; ^4^ Interdisciplinary Centre for Advanced Materials Simulation (ICAMS) Ruhr‐Universität Bochum 44780 Bochum Germany; ^5^ Department of Chemistry and Materials Science Aalto University Kemistintie 1 02150 Espoo Finland; ^6^ Université Grenoble Alpes Université Savoie Mont Blanc CNRS Grenoble INP LEPMI Grenoble 38000 France; ^7^ Catalysis Research Center TUM Ernst‐Otto‐Fischer‐Str. 1 85748 Garching Germany

**Keywords:** electrochemical scanning tunneling microscopy, molecular dynamics simulations with machine learning potential, online inductively coupled plasma mass spectrometry, palladium hydride formation, proton electroreduction, strain relaxation, surface reconstruction

## Abstract

Designing electrocatalysts with optimal activity and selectivity relies on a thorough understanding of the surface structure under reaction conditions. In this study, experimental and computational approaches are combined to elucidate reconstruction processes on low‐index Pd surfaces during H‐insertion following proton electroreduction. While electrochemical scanning tunneling microscopy clearly reveals pronounced surface roughening and morphological changes on Pd(111), Pd(110), and Pd(100) surfaces during cyclic voltammetry, a complementary analysis using inductively coupled plasma mass spectrometry excludes Pd dissolution as the primary cause of the observed restructuring. Large‐scale molecular dynamics simulations further show that these surface alterations are related to the creation and propagation of structural defects as well as phase transformations that take place during hydride formation.

## Graphical abstract

Optimizing electrocatalyst performance requires insights into surface and subsurface structures under reaction conditions. This study uncovers hydrogen‐induced reconstruction processes on low‐index palladium (Pd) surfaces during proton electroreduction by integrating computational and experimental approaches. The results reveal that surface roughness and morphological changes arise from hydrogen‐induced hydride formation and subsequent reconstruction to minimize strain in PdH_x_/Pd‐interfaces, and not from surface dissolution.

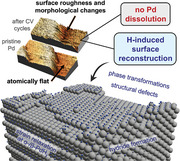

## 1 Introduction

The surface morphology of catalytic materials influences their performance, reaction kinetics and selectivity. Understanding and controlling surface structures throughout the catalyst's lifetime is thus crucial for optimizing the overall catalytic activity. Surfaces of most metal catalysts undergo various atomic‐scale relaxations and reconstructions that depend on the chemical bonding of interacting species and the electrochemical environment established by electrolyte and applied electrode potential.^[^ ^1^, ^2^, ^3^, ^4^ ^]^ Hydrogen‐induced reconstruction has been studied extensively for metal surfaces in electrocatalytic reactions.^[^ ^5^, ^6^, ^7^ ^]^ Palladium (Pd), a noble transition metal known for catalyzing hydrogen‐based reduction reactions,^[^ ^8^, ^9^ ^]^ is particularly well‐studied in this context.^[^ ^10^, ^11^, ^12^, ^13^ ^]^

Previous studies revealed that high hydrogen coverage induces a reconstruction of the least stable low‐index Pd(110) surface, leading to formation of pairing‐row or missing‐row structures.^[^ ^14^, ^15^, ^16^, ^17^, ^18^, ^19^ ^]^ First‐principles calculations based on density functional theory (DFT) suggest that migration of Pd atoms and subsequent reconstructions arise due to interactions with diffusing hydrogen atoms.^[^ ^20^ ^]^ Underlying mechanisms of these reconstructions can nowadays be observed in situ even at low hydrogen coverage using scanning tunnelling microscopy (STM).^[^ ^19^ ^]^ For instance, Kralj et al. demonstrated that the reconstruction of Pd(110) surfaces is an autocatalytic process driven by the hydrogen adsorption.^[^ ^21^ ^]^ However, the majority of experimental work has been conducted under ultra‐high vacuum conditions, while studies on H‐induced reconstructions in aqueous electrolytes under electrochemical conditions remain rather scarce.

Recently, we investigated variations of the surface morphology of Pd(111), Pd(100), and Pd(110) surfaces and their impact on the hydrogen evolution reaction activity in aqueous electrolytes using a combination of electrochemical scanning tunneling microscopy (EC‐STM) and DFT calculations.^[^ ^22^ ^]^ This study confirmed that the surface alteration are induced by the absorption of hydrogen into the bulk during potential cycling, but phenomena related to subsurface hydride formation that induces local plastic deformation, surface phase transitions, and nucleation of extended defects, such as stacking faults and misfit dislocations, remained unresolved.

The present study combines several complementary experimental and computational approaches to elucidate the restructuring processes on Pd(111), Pd(110), and Pd(100) surfaces on larger time and length scales. Herein, we employ EC‐STM to observe H‐induced morphological changes of these surfaces, on‐line dissolution measurements using a scanning flow cell in combination with an inductively coupled plasma mass spectrometer (SFC‐ICP‐MS) to quantitatively assess Pd dissolution during H ingress, and large‐scale molecular dynamics (MD) simulations, enabled by advancements in machine‐learning interatomic potentials,^[^ ^23^, ^24^, ^25^, ^26^ ^]^ to follow the initial stages of surface roughening associated with formation of extended defects and plastic deformation.

The SFC‐ICP‐MS measurements confirm no Pd dissolution from single‐crystal Pd surfaces, attributing the increased surface roughness and morphological changes observed by EC‐STM solely to H‐induced surface reconstruction. The MD simulations reveal that dynamic rearrangements and restructuring of the Pd surfaces emerge after surface and subsurface hydride formation. Additionally, they underscore the importance of structural defects and phase transformations, as well as their influence on the mobility of Pd and H atoms. These insights enhance our understanding of Pd hydride reconstructions in aqueous electrolytes that are crucial for the design of highly active and stable Pd‐based electrocatalysts.

## 2 Results and Discussion

### 2.1 Activity Degradation of Pd(hkl) Surfaces during Hydrogen Evolution Reaction (HER)

**Figure 1:**
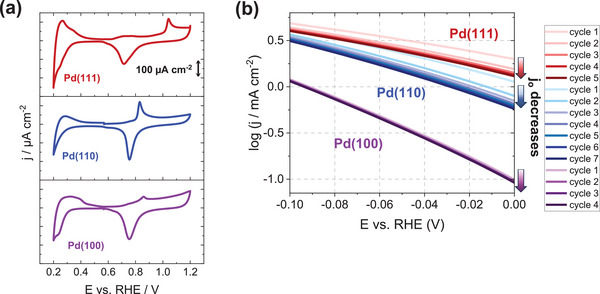
Characteristic CVs of Pd single crystals and HER activity degradation. a) CVs of annealed Pd single crystals (Pd(111), Pd(110) and Pd(100)) in Ar‐sat. 0.1 m HClO_4_ recorded with 50 mV s^−1^ scan rate. b) Degradation of HER activity with increasing cycle numbers, shown by Tafel plots (cathodic scan) for the respective Pd crystals in H_2_‐saturated 0.1 m HClO_4_, recorded with 50 mV s^−1^ scan rate.

Cyclic voltammetry (CV) was used to investigate surface changes of Pd(111), Pd(110), and Pd(100) single crystals. Their well‐ordered structure was confirmed by recording CVs in Ar‐saturated 0.1 m HClO_4_, in the potential range from 0.2 to 1.2 V versus reversible hydrogen electrode (RHE), as displayed in **Figure** **1**a.^[^ ^27^, ^28^ ^]^ It must be noted that H‐induced morphological changes were investigated with freshly annealed single crystals within the potential range of ‐0.18 V to 0.52 V versus RHE. Cathodic potentials up to ‐0.18 V promote hydride formation, which starts at potentials smaller than the hydrogen underpotential deposition (H_UPD_) potential region for bulk Pd surfaces.^[^ ^22^ ^]^ Importantly, we avoid potential ranges that may lead to additional morphological alterations, such as those from Pd dissolution or similar to roughening observed for Pt single crystals.^[^ ^29^, ^30^, ^31^, ^32^ ^]^ Additionally, the single crystals were not subjected to large anodic potentials to avoid surface oxidation. Figure 1b shows Tafel plots, revealing activity trends for the three Pd single crystals. However, our focus shifts from activity comparison, already addressed in our previous work,^[^ ^22^ ^]^ to activity degradation along with increasing cycling numbers. Notably, Pd(111) and Pd(110) exhibited rapid activity declines after five and seven cycles, respectively. Moreover, Figure S1 (Supporting Information) provides optical microscopy images for the post‐HER cycling of all investigated single crystals. For Pd(100), despite initial stability, optical microscopy (10x magnification) after four cycles revealed significant morphological surface changes, including hills, grooves, and dimples.

### 2.2 In Situ Investigation of Morphological Changes at Pd(hkl) Surfaces

**Figure 2:**
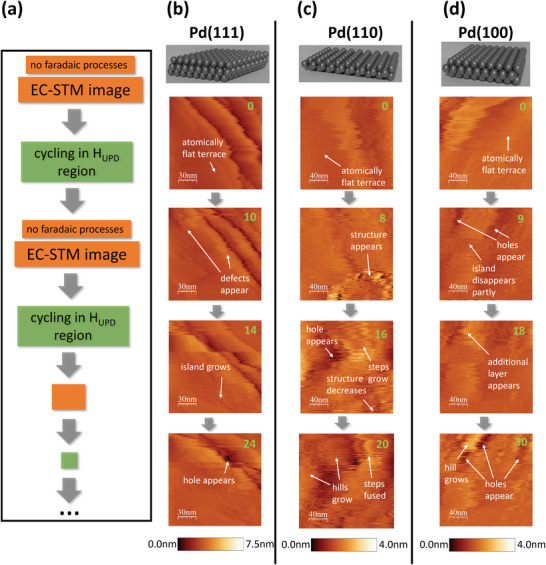
EC‐STM images highlighting morphological changes of Pd single crystal surfaces during potential cycling in the H_UPD_/double‐layer potential region. a) Schematic illustration of the EC‐STM measuring procedure to explore H‐induced morphological changes on Pd single crystals. Selected EC‐STM images displaying the morphological changes on Pd single crystal surfaces; b) Pd(111), c) Pd(110), and d) Pd(100) after varied numbers of CV cycles in the H_UPD_/double‐layer potential region. During image acquisition, an electrochemical neutral potential within the double layer was set. The number of completed CV cycles before capturing the EC‐STM image is highlighted in green in the top right corner of each image.

EC‐STM permits visualizing gradual morphological changes at Pd single crystal surfaces during electrochemical cycling. As illustrated in **Figure** **2**a, after each cycling process within the H_UPD_/double‐layer potential region, we systematically recorded 150 nm x 150 nm EC‐STM images utilizing a constant tip and sample potential in the double‐layer region. A detailed description of all experimental parameters can be found in Tables S1 and S2 (Supporting Information). Figure 2b–d displays selected EC‐STM surface images after the respective executed number of CV cycles on freshly annealed Pd(111), Pd(110), and Pd(100) single crystals. An overview of all intermediate surface snapshots and representative CVs captured during the cycling procedure are provided in Figures S2–S4 (Supporting Information). The first EC‐STM images (cycle 0) of the pristine Pd single crystals exhibit atomically flat terraces, confirming successful annealing procedures. As the cycling progresses, multiple‐atomic structural defects (holes, steps, islands) emerge at terraces and in areas adjacent to step edges for Pd(111). Those defects are distinguishable by their lighter color compared to that of the smooth areas of the surface terraces. This color contrast is accentuated due to a larger range of the color bar for Pd(111) compared to those of the other terminations. For Pd(110), a complex surface structure appeared after eight cycles at the bottom of the image, reaching up to more than 100 nm in lateral size. However, with continued cycling, the structure gradually diminished, giving rise to nearby holes and hills. For Pd(100), the EC‐STM image of the pristine surface (0 cycles) revealed the presence of an island, for which a roughly ≈1500 nm^2^ area partially disappeared after nine potential cycles. Furthermore, adlayers and holes emerged on top and adjacent to the initial island, which coalesced into valleys and hills after thirty potential cycles. In summary, islands gained both in lateral size and height, reaching up to several tenths of nanometers, with an increasing number of cycles for all investigated surface orientations.

**Figure 3:**
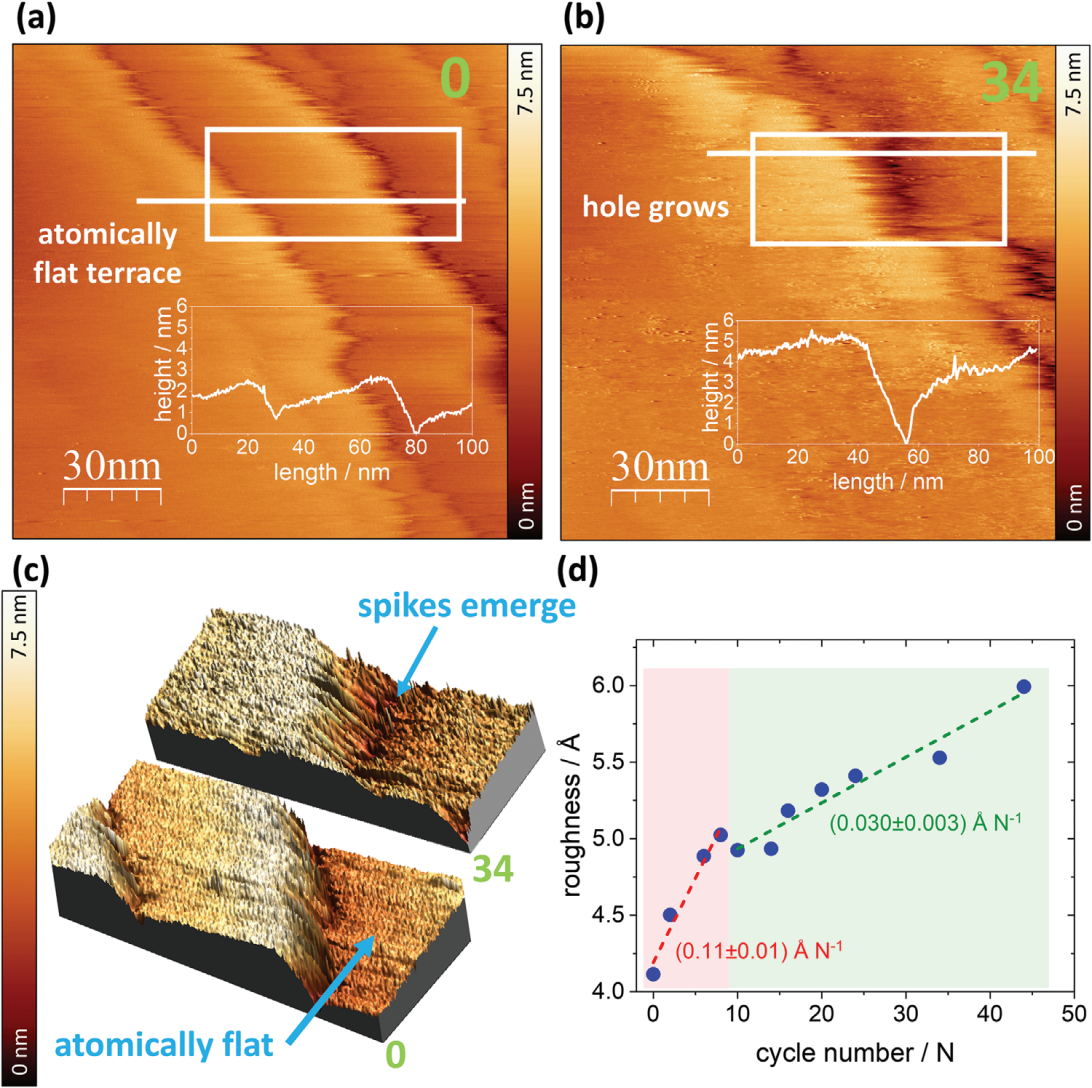
Roughness analysis of Pd(111) after potential cycling in the H_UPD_/double‐layer region. EC‐STM image of the a) pristine Pd(111) surface and b) after 34 CV cycles. The white line indicates the position of the height profile shown in the insets. c) 3D EC‐STM visualization of 80 nm x 40 nm regions highlighted by the white rectangles in the 2D images above. d) Roughness analysis results for the Pd(111) surface, illustrating two distinct patterns of roughness development over the CV cycles.

For a more detailed analysis, we turn our attention specifically to Pd(111) and compare its morphology after thirty‐four cycles to the freshly annealed surface, as displayed in **Figure** **3**. Despite a minor sample drift, we successfully maintained a comparable surface position for both images. The height profile, derived from a line scan at a position marked by a white line in the images, is plotted in the insets. Additionally, 3D visualizations obtained from the line scans are displayed in Figure 3c, extracted from the 80 nm x 40 nm areas specified by the white rectangles in the 2D images in Figure 3a,b. The EC‐STM image of the pristine surface exposes two relatively high Pd steps with heights of 1.6 and 2.8 nm, respectively, and an atomically flat terrace in between. The two steps converge during cycling, leading to the formation of a hole (≈5 nm deep) mentioned above, located in the vicinity of the second step. Apart from the hole formation, the 3D visualization reveals the appearance of a large number of conspicuous spikes, underscoring a substantial increase in surface roughness at the Ångström level. The density and height of these spikes differ across different surface regions. Particularly, the areas adjacent to the steps and within the hole display more pronounced spike density and height compared to the terraces. To quantitatively assess this change in surface roughness, we conducted a detailed analysis of the EC‐STM images of Pd(111) using a modified version of the root‐mean‐squared roughness.^[^ ^33^ ^]^ Our roughness calculation method reduces the influence of nanometer‐sized morphological features, such as islands, holes, or steps, and instead accentuates the surface roughness at sub‐nanometer scales along those features. A more detailed description of the roughness analysis is provided in Section S1 (Supporting Information). Figure 3d depicts the calculated roughness increase along the potential cycling of the Pd(111) surface. However, while EC‐STM reveals morphological changes on the Pd surface, addressing the nature of this alteration remains a challenge. Generally, the formation of ad‐atoms and ad‐islands might be attributed to surface dissolution under potential control coupled with subsequent redeposition and/or H‐induced surface reconstruction.

### 2.3 Investigation of Pd Dissolution from Pd(hkl) Surfaces and Pd Nanoparticles

**Figure 4:**
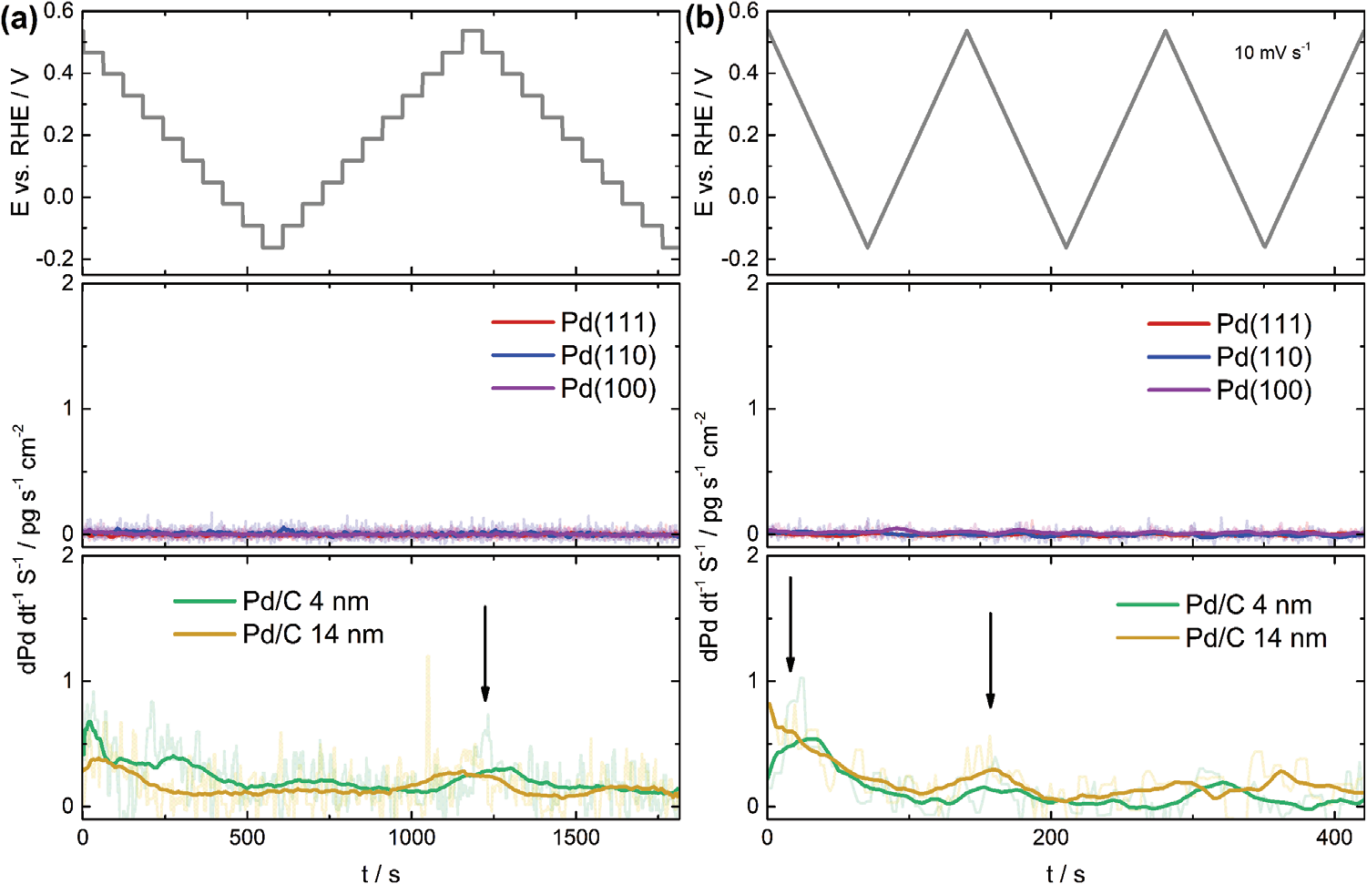
On‐line dissolution experiments for Pd(hkl) surfaces and Pd nanoparticles. Electrochemical protocols (top panels) and dissolution profiles for the three Pd single‐crystal surfaces (middle panels) and Pd/C nanoparticles with an average size of 4 and 14 nm (lower panels) in Ar‐saturated 0.1 m HClO_4_ for a) the potential‐step protocol and b) the cyclic voltammetry protocol. The black arrows indicate the observed dissolution peaks in the case of the measurements with nanoparticles.

On‐line dissolution measurements with the SFC‐ICP‐MS technique were performed to investigate if the restructuring of the surface observed by EC‐STM is caused partly by the dissolution of Pd atoms. First, a potential‐step protocol emulating the EC‐STM experiments was carried out for the single crystals. The results presented in **Figure** **4**a show no dissolution for all surface planes. To exclude a spurious influence of short finite potential jumps in the protocol, a second potential sweep protocol consisting of successive CV measurements was also carried out. As displayed in Figure 4b, no dissolution could be detected in this case as well.

Viola et al. measured dissolution from commercial 4 nm sized Pd/C nanoparticles using a cycling protocol with 0.03 and 0.60 V versus RHE as lower potential limit (LPL) and upper potential limit (UPL), respectively.^[^ ^34^ ^]^ In this case, dissolution lower than 0.003% of the total Pd mass was detected. Since no dissolution was measured for Pd single‐crystal electrodes, additional measurements were performed with Pd nanoparticles to address this discrepancy. First, analogous experiments to the work presented by Viola et al. were performed here with the same 4 nm nanoparticles and also with larger Pd/C nanoparticles of 14 nm. Small dissolved amounts were also detected using the current experimental setup, with higher dissolution for the 4 nm Pd/C nanoparticles (see Figure S5, Supporting Information). The dissolution measured here is probably due to anodic dissolution at potentials close to the UPL (ca. 0.5 V vs RHE) and therefore the tailing of the dissolution signal is still observed during the reverse scan. These results confirm the reliability of the online dissolution experimental setup used in this work. Pizzutilo et al. detected higher dissolution for a Pd/C nanoparticle sample than for polycrystalline Pd, with an onset potential of dissolution that seems to be in line with the signal measured in this work, although an UPL of 0.9 V versus RHE and a slower scan rate (2 mV s^−1^) were employed in their electrochemical protocol.^[^ ^35^ ^]^ The size‐dependent dissolution observed in the present work is in line with the fact that the smaller nanoparticles are less stable than extended surfaces, as described by the Kelvin equation (also known as Gibbs‐Thomson equation in electrochemistry), according to which there is a decrease in the onset potential of dissolution/deposition by an amount inversely proportional to the radius of the nanoparticle.^[^ ^36^, ^37^, ^38^, ^39^ ^]^ For a direct comparison with the dissolution experiments for Pd single crystals shown in Figure 4, the same protocols were also carried out for the nanocrystals. As shown in Figure 4a, a slight dissolution is detected for the potential‐step protocol at potential values close to the UPL. Only very small dissolution peaks can be observed for the cyclic voltammetry protocol (Figure 4b), also once the maximum of 0.52 V versus RHE is reached. In both cases, the dissolution rates are very low and, therefore, the exact correspondence between the electrochemical signal and the dissolution profile is difficult to determine because it can be greatly affected by the residence times of the dissolved ions near the surface. However, it is clear that the dissolution detected here is related to the low‐extent oxidation of the Pd surface near the UPL and it does not occur at potentials where the hydrogen insertion/de‐insertion processes take place. The detection limits obtained for the present online dissolution measurements (including all Pd single crystal and nanoparticle experiments) are in the order of 10^−4^ ng s^−1^ cm^−2^ (see Figure S6, Supporting Information). The latter number corresponds to less than 0.1% of a monolayer in all cases, considering the time scales of the experiments. All the above observations rule out Pd dissolution/redeposition as the primary cause of the morphological changes observed with EC‐STM.

### 2.4 Atomistic Mechanisms Underlying the H‐Induced Pd Surface Reconstruction

**Figure 5:**
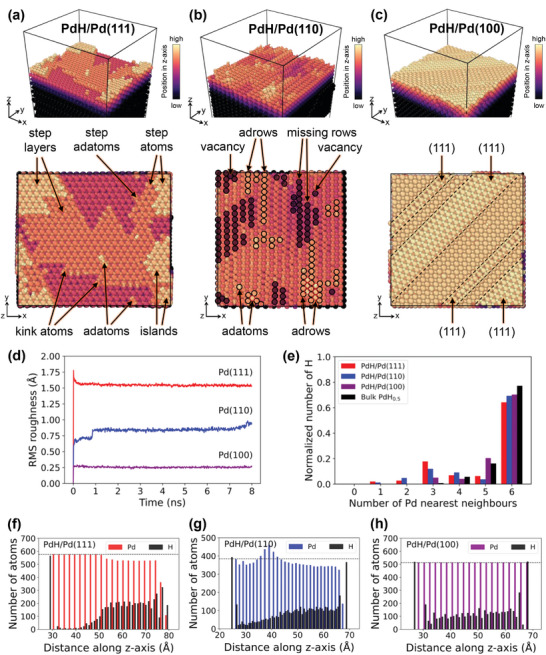
Surface analysis of the equilibrated structures obtained from MD simulations. The reoptimized structures after 8 ns MD at 300 K with color gradient indicating the atomic positions along the *z* direction for a) PdH/Pd(111), b) PdH/Pd(110), and c) PdH/Pd(100), d) Calculated root mean square (RMS) roughness for the three Pd surfaces as a function of simulation time, e) Number of H atoms surrounded by different numbers of Pd nearest neighbors for the three reoptimized structures after 8 ns MD, which is normalized by the total number of H in each system. The nearest neighbor analysis for bulk PdH_0.5_ is also presented for comparison; f–h) Distributions of Pd and H atoms along the *z* direction (surface normal), where dashed lines indicate the number of Pd per layer in the initial structures.

As the online SFC‐ICP‐MS measurements reveal no Pd dissolution during the excursions into negative potential values, the increased surface roughness and morphological changes observed by EC‐STM should be attributed solely to H‐induced surface reconstruction. To unravel the atomic‐scale mechanisms governing surface restructuring processes during hydride formation, large‐scale MD simulations were performed for PdH/Pd(111), PdH/Pd(110), and PdH/Pd(100) slab models (see details in Section 4, Experimental section—MD simulations and Section S3, Supporting Information). The surface structures after 8 ns MD equilibration at 300 K are shown in **Figures** **5**a–c and S8 (Supporting Information). The simulations reveal a formation of various defects (specified in detail in Figure 5a–c) that lead to surface restructuring and roughening for all studied surfaces, in accordance with the EC‐STM observations. The roughening occurs already during the first ns for all three surfaces (Figure 5d), with roughness values of ≈1.53 Å, ≈0.94 Å, and ≈0.27 Å at 8 ns for the Pd(111), Pd(110), and Pd(100) surfaces, respectively. The rapid surface roughening is due to a high initial concentration of hydrogen in the subsurface layers of our Pd model system, as discussed in Section 4, Experimental Section—MD simulations. We deliberately started the simulations with large amounts of hydrogen absorbed in several subsurface layers to capture efficiently surface roughening processes associated with β‐PdH_x_ hydride formation. While generally possible, modeling the entire process of hydrogen ingress into a pristine (flat) Pd surface during electrochemical charging would require significantly longer simulation times. To validate that a substantial amount of hydrogen in the subsurface layers is essential to induce surface roughness, we carried out additional simulations for the Pd(111) surfaces with varying number of hydrogen sublayers (Figure S9, Supporting Information). These simulations demonstrate that significant surface roughening starts only when the thickness of β‐PdH_x_ surface hydride exceeds four hydrogen layers. The highest roughness observed for the Pd(111) surface is likely related to its close‐packed structure and higher coordination number compared to the more open structures of the (110) and (100) surfaces.^[^ ^40^ ^]^ This can also be attributed to the observed lowest surface formation energy of the PdH/Pd(111) system after MD equilibration (see Figure S10, Supporting Information). The PdH/Pd(111) surface is roughened by formation of adatoms, islands, and two step layers (Figure 5a). In contrast, two new Pd adlayers form on PdH/Pd(110) and this surface undergoes a missing‐row reconstruction accompanied by formation of adrows, adatoms, and vacancies (Figure 5b). It is worth noting that among the low‐index surfaces, the H‐containing Pd(111) is the most stable surface.^[^ ^41^ ^]^ Moreover, the H‐covered missing‐row reconstructed surface of PdH/Pd(110) is more stable than the ideal one due to the exposure of the (111) facet.^[^ ^42^ ^]^ This explains the observed missing‐row reconstruction of Pd(110) during hydride formation.^[^ ^13^, ^20^, ^43^ ^]^ In case of PdH/Pd(100), the surface roughening is induced by lifting some surface atoms and formation of (111) facets (Figure 5c). This indicates that also the Pd(100) surface tends to transform into the most stable (111) termination upon hydride formation.

**Figure 6:**
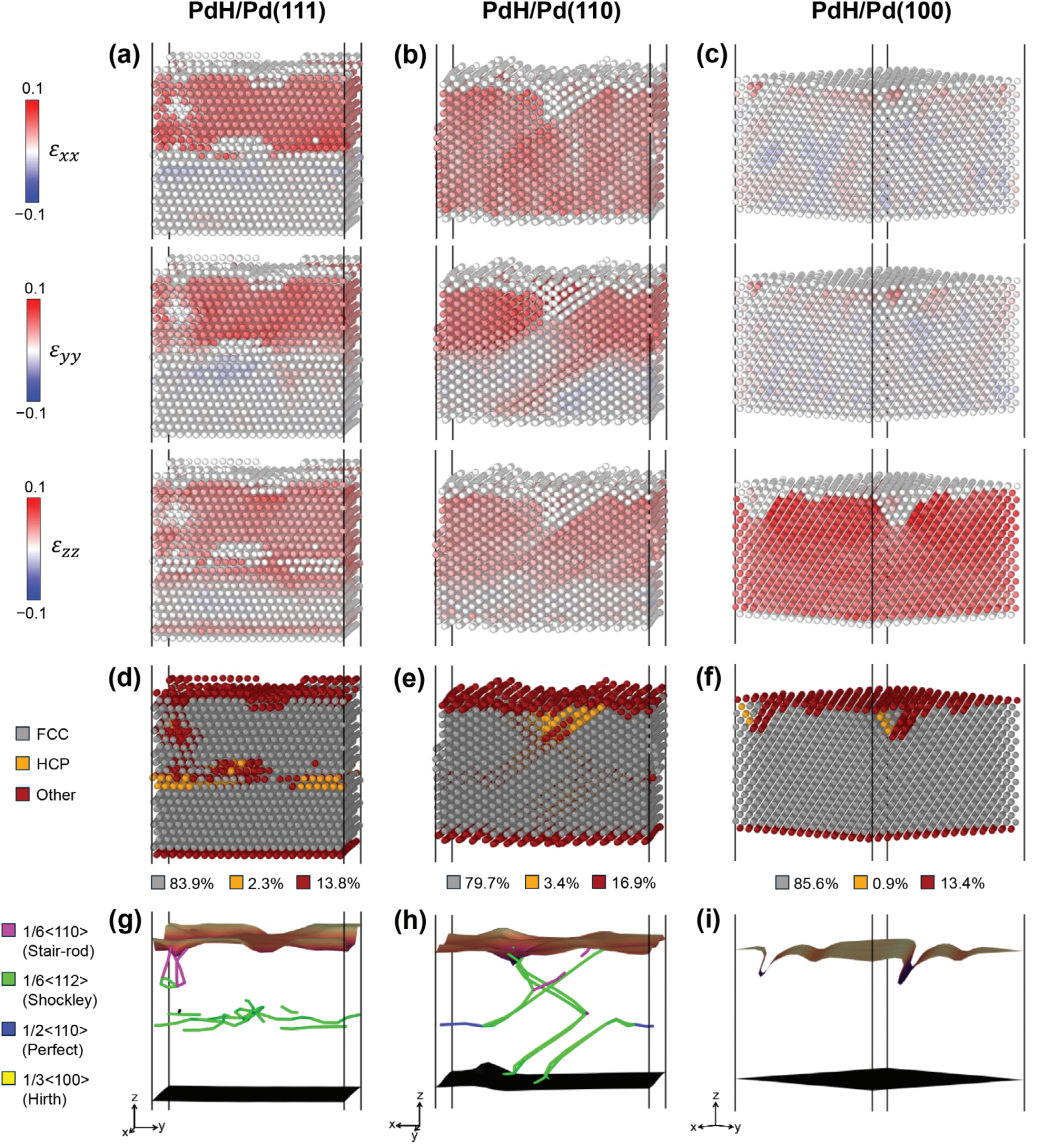
Strain and dislocation analysis of the equilibrated structures obtained from MD simulations. The elastic strain (𝜀) for each Pd atom along the *x*‐, *y*‐, and *z*‐axis (H atoms are omitted for clarity) of the reoptimized structures after 8 ns MD at 300 K for a) PdH/Pd(111), b) PdH/Pd(110), and c) PdH/Pd(100), evaluated by determining the total deformation of the crystal around each atom, with respect to an fcc Pd with a lattice constant of 3.891 Å, where positive and negative values indicate the tensile and compressive strains, respectively; d–f) visualization of the local structures and g–i) dislocations in these model systems.

The surface roughening was accompanied by additional processes, including H_2_ formation, H diffusion, and H ad‐ and absorption (see Section S4, Supporting Information). We observed that the absorbed H occupies mostly the octahedral interstitial sites in the Pd matrix for all studied surfaces (see Figure 5e and Section S5, Supporting Information). Furthermore, the distributions of Pd and H atoms along the *z* direction (surface normal) were evaluated for all surfaces (see Figure 5f–h and Section S6, Supporting Information). As displayed in Figure 5f–h, while the number of Pd atoms in each layer of the (100) surface remains the same as in the initial structure, it varies for the (111) and (110) cases. This corroborates the larger roughness observed in the (111) and (110) surfaces compared to the (100), which is caused by the transport of some subsurface Pd atoms to the surface. Despite distinct H distributions in the interior of the slabs, there is a clear preference for the H atoms to stay on the surface for all three terminations. This aligns well with previous computational studies demonstrating stronger H adsorption on surfaces compared to absorption in subsurface interstitial sites.^[^ ^41^, ^44^, ^45^ ^]^ It should be noted that the bottom surface of the slab was populated mainly by H atoms desorbed from the top surface which travelled to the bottom surface due to periodic boundary conditions. The final H distributions in the three slabs at the end of MD runs are clearly different. The Pd(111) slab retains a rather sharp interface between the β‐phase (hydride‐rich) in the top half of the slab and the α‐phase (hydride‐poor) in the bottom half. In contrast, the Pd(110) and especially Pd(100) slabs develop more uniform H distributions throughout the whole thickness. Furthermore, as shown in **Figure** **6**d–i, the three equilibrated structures also exhibit markedly different internal morphologies. These contrasting outcomes originate from a complex interplay between residual stresses and strains, the character of extended defects (in particular misfit dislocations), and H diffusion.

It is well known that the lattice constant of bulk Pd increases when it absorbs H and the formation of bulk Pd hydride results in a large lattice expansion.^[^ ^46^ ^]^ The initial structures in our simulations were relaxed, but their lateral dimensions were kept fixed (to those of the equilibrium fcc Pd lattice) to mimic the existence of a thick Pd substrate that does not undergo any large elastic deformation. As a result, the initial subsurface hydride layers were subject to compressive lateral stresses and underwent a lattice expansion in the direction normal to the surface upon the initial relaxation. During the subsequent MD simulations, there was a large driving force to further release the lateral residual stresses that led to the observed surface reconstruction and roughening, as well as a formation of lattice defects, such as dislocations and stacking faults, inside the slabs. A fundamental analysis of principal strains and of the observed defect types is shown for all three systems in Figure 6; a further analysis of H distributions, anisotropic H diffusion, and defect structures is provided in Sections S6–S8 (Supporting Information).

Specifically for PdH/Pd(111) (see Figure 6a), there exist large tensile strains (note that the strains were computed with respect to the equilibrium fcc Pd lattice) along all three principal directions in the top‐half of the slab where the β‐hydride phase is located (compare to Figure 5f). These strains are consistent with the lattice expansion of the β‐hydride phase. Interestingly, the boundary between the strained top and unstrained bottom halves is almost atomically sharp. As shown in Figure 6d,g and Figures S15 and S17 (Supporting Information), the reason for this abrupt change is a planar network of interfacial dislocations that accommodate the lattice misfit at the β‐hydride(top)/α‐hydride(bottom) interface. These dislocations are common Shockley partial dislocations occurring on (111) close‐packed planes in fcc metals.^[^ ^47^, ^48^, ^49^ ^]^ For the PdH/Pd(111) geometry, the dislocation network thus can form readily at the interface. Additionally, the network of these dislocations leads to local changes of the A‐B‐C stacking of the fcc {111} planes into the hexagonal A‐B‐A stacking at the interface. All these factors contribute to the stability and the narrow width of the β‐hydride/α‐hydride interface. These results are consistent with recent simulations^[^ ^50^ ^]^ as well as experiments on Pd nanoparticles^[^ ^51^ ^]^ that indicate that the misfit dislocations hinder the propagation of the phase boundary by slowing down the H diffusion.

For PdH/Pd(110), the initial sharp interface gradually broadens during the MD run, but the difference between the top and bottom halves of the slab remains discernible, with H concentration remaining higher in the original hydride and decaying gradually into the Pd matrix (compare Figures 6b and 5g). The initial strain in the slab is relieved again by formation of lattice dislocations, but these are positioned diagonally throughout the slab following the geometrical inclination of the (111) planes (Figure 6e,h and Figure S17, Supporting Information). This is likely the primary reason why the mobility of H atoms is affected much less than in PdH/Pd(111). The tensile strains along the *y* and *z* directions (both <110>) are localized mostly in the upper‐half sublayers, where the H concentration remains higher (compare Figures 6b and 5g). The tensile strain along the *x* direction (<100>) is distributed uniformly over the whole slab, meaning that also the original Pd substrate becomes strained. The reason is that one of the dislocations propagated through the slab to the bottom surface and effectively removed one layer of Pd atoms. It should be noted that the dislocation activity (not shown) was rather rich throughout the whole simulation. In the initial few ns, it was mostly confined to the hydride phase, but eventually it encompassed the whole slab, thus continuously accommodating the stresses that arise due to redistribution of H.

Interestingly, for PdH/Pd(100) we did not observe any formation of dislocations (Figure 6i) and the whole slab only expanded along the surface normal, eventually reaching an almost homogeneous H distribution throughout the whole thickness (Figure 5h). Correspondingly, a large tensile strain occurs only in the *z* direction, normal to the surface, and there are only very small strains along the *x* and *y* directions (Figure 6c). Only a small number of atoms with altered coordination exist near the surface to accommodate the rearrangement of some surface atoms into the (111) orientation (Figure 6f). Our results are consistent with studies of {100} oriented cubic nanoparticles where suppression of extended defects and coherent phase boundaries were reported as well.^[^ ^52^ ^]^ It should be noted that all microscopic phenomena observed in our simulations have been debated actively in connection with H‐induced phase transformations and H absorption dynamics in Pd nanocrystals and thin films.^[^ ^50^, ^51^, ^53^, ^54^, ^55^, ^56^, ^57^, ^58^, ^59^, ^60^ ^]^ These studies demonstrated the possibility of both a fully coherent metal‐hydride transformation and a transformation accommodated by incoherent interface between the hydride phases, depending on the system size, geometry and morphology as well as hydrogen charging.

## 3 Conclusion

A gradual roughening of the Pd(111), Pd(110), and Pd(100) surfaces induced by potential cycling due to proton reduction was observed using EC‐STM. Online SFC‐ICP‐MS measurements detected no Pd dissolution for all investigated surface orientations as well as for nanoparticles at potentials within the hydrogen underpotential deposition and insertion region. This indicates that the observed roughness and morphological changes of the Pd surfaces arise solely from the H‐induced reconstruction. Through MD simulations, we have shed light on the dynamic rearrangement and restructuring of Pd surfaces after surface and subsurface hydride formation. These restructuring processes are associated with creation of structural defects and phase transformations within the subsurface layers. This combined study provides both experimental and theoretical insights into the dynamics of electrocatalyst surface reconstruction under reaction conditions, with a special focus on Pd hydride formation. Understanding these H‐induced reconstruction processes of Pd surfaces is an indispensable prerequisite for the design of highly active and selective electrocatalysts.^[^ ^61^, ^62^, ^63^, ^64^, ^65^ ^]^

## 4 Experimental Section

### Palladium Single Crystal Preparation and Electrochemical Characterization

For the conducted experiments, Pd(111) (Ø 6 mm, 99.999%, MaTecK, Jülich, Germany), Pd(111), Pd(110), and Pd(100) single crystal (Ø 5 mm, 99.999%, MaTecK, Jülich, Germany) disc electrodes were employed with a surface roughness below 0.03 µm and an orientation accuracy greater than 0.1°. To perform atomically accurate and contamination‐free STM measurements, a thorough cleaning and annealing process of the single crystals is necessary before conducting the measurements. All glassware, Teflon pieces, and measurement cells were previously cleaned with “Caro's Acid,” a 3:1 mixture of sulfuric acid (96% H_2_SO_4_ Suprapur, Merck, Germany) and hydrogen peroxide (30% H_2_O_2_, Suprapur, Merck, Germany). After cleaning with the acid, all pieces were washed multiple times with boiling ultrapure water (Evoqua Milli‐Q (18.2 MΩ cm)). The electrochemical cleaning of the single crystals was conducted in a classical single‐crystal cell consisting of a three‐electrode setup connected to a VSP‐300 potentiostat (Bio‐Logic, France). For the electrolyte, 70% perchloric acid (Suprapur, Merck, Germany) was diluted with ultrapure water to obtain a 0.1 m concentration. The employed 0.1 m HClO_4_ electrolyte was purged with Ar (Westfalen, Germany) for 30 min. The counter electrode (CE) and reference electrode (RE) consist of a curled polycrystalline Pd‐wire (Ø = 0.25 mm, 99.95%, MaTecK, Jülich, Germany) and a Mercury‐Mercurous Sulfate (MMS) RE (B 3610+ from SI Analytics), respectively. Before EC‐STM experiments, the cleaning procedure relies on conducting CVs (50 mV s^−1^) in a potential range from 270 mV to 1270 mV versus RHE. Subsequently, the respective crystal is annealed with an inductive heater (20–80 kHz, 15 KW– EQ‐SP‐15A, MTI, USA) to obtain a well‐oriented surface. Annealing occurs in a continuously Ar‐flushed glass vessel, where the Pd single crystals are mounted on a Pd wire in order to avoid contact with impurities. The annealing temperature corresponds roughly to 800–900 °C for 60 s, followed by a cooling step at room temperature for 5 min. This procedure of annealing and cooling was repeated multiple times, however, with decreasing annealing times of 60, 50, 40, and 30 s, respectively. After the annealing procedure, the Ar‐containing glass vessel containing the Pd crystal was transferred into a vacuum‐proof chamber containing an Ar atmosphere before being inserted in the glovebox (Ar atmosphere), in which all EC‐STM experiments were conducted, or in the SFC‐ICP‐MS setup for the on‐line dissolution experiments. For the electrocatalytic experiments, the single crystals were annealed with a Heraeus Instruments RO 7/50 furnace. A detailed description of the annealing procedure, as well as the procedure for CV and activity measurements on the single crystals can be found in our previous study.^[^ ^22^ ^]^

### EC‐STM

All EC‐STM measurements were conducted using a Multimode Nanoscope V SPM from Bruker connected to a Bruker Nanoscope Universal bipotentiostat and a Nanoscope V controller, located inside a glovebox (GS ALPHA X‐Line) filled with Ar (5.0, Westfalen, Germany) gas. After preparation, the respective single crystals were mounted in a self‐made, waterproof sample holder basically consisting of a conductive base plate and a Teflon ring to avoid any leaking electrolyte. STM tips used in the experiments consist of a Pt–Ir alloy with 80% Pt and 20% Ir (Ø = 0.25, 99.9+%, MaTecK), which were produced by a mechanical cutting and pulling procedure.^[^ ^66^ ^]^ Before EC‐STM experiments, each tip was isolated by Apiezon Wax^[^ ^67^ ^]^ in order to reduce the exposure of Pt–Ir side surfaces, which could significantly contribute toward the faradaic current that would even exceed the tunneling current in case of too‐large interfaces. For the CE, a curled Pd wire (Ø 0.25 mm, 99.95%, MaTecK, Germany) was employed, while for the RE, a self‐made Silver‐Silver Chloride (SSC) RE‐setup was developed, which is partially immersed in the center of the miniature cell of STM head. During all measurements, a Nano20 active insulation plate from Accurion is mounted beneath the STM to reduce vibrational artifacts in the recorded data. The experiments aim to investigate and visualize the H‐induced morphology modification of the low‐indexed basal planes Pd(111), Pd(100), and Pd(110). Therefore, reference STM images of the Pd crystals were captured from the freshly annealed or initial state of the material with applied potentials in the double‐layer region, in which the absence of faradaic processes was previously validated. An overview of the respective potential ranges, applied during cycling, and the parameters chosen for the respective EC‐STM images, including sample and tip potential, scan size and rate, and integral and proportional gain, can be found in Table S1 (Supporting Information). Afterwards, a series of CVs were conducted within the specified potential region to initiate H‐induced morphology changes. This potential range contains the H_UPD_ and the double‐layer region for the respective Pd single crystals in 0.1 M HClO_4_. In order to ensure uniform morphology changes across all single crystals, the potentials were carefully chosen and adjusted during the potential cycling so that the current densities across different single crystals are roughly comparable. Table S2 (Supporting Information) provides a list of the parameters selected for the respective CVs. It is highlighted that EC‐STM images were recorded at stationary conditions, which means not during the potential cycling. Therefore, the same double‐layer region potential used during the imaging of each pristine Pd single crystal was re‐applied before capturing another EC‐STM image. Despite our efforts to maintain the same tip location, it proved to be challenging due to the artificial noise and thermal drift arising over time during EC‐STM experiments.

### Palladium Nanoparticles Supported on Vulcan XC‐72

For Pd/C nanoparticles with an average diameter of 4 nm, Pd/Vulcan XC‐72 catalyst with a nominal Pd weight fraction of 20% was purchased from Premetek (item P30A200) and used as received. See Section S9 (Supporting Information) for additional characterizations. For Pd/C nanoparticles with an average diameter of 14 nm, Pd nanoparticles were synthesized using a two‐microemulsion technique, as detailed in the work by Moumaneix et al.^[^ ^68^ ^]^ Briefly, two separate microemulsions were prepared, one containing a Pd precursor (Pd(NH_3_)_4_Cl_2_, 0.1 m) and the other a reducing agent (hydrazine hydrate, Sigma‐Aldrich, approx. 64%), both supplemented with the same amount of surfactant (AOT, Sigma‐Aldrich, ≥ 97%) and iso‐octane (Sigma‐Aldrich, ≥ 99%). Following thorough stirring, the microemulsions were combined to yield Pd nanoparticles. Subsequently, a carbon support (Vulcan XC‐72, Cabot) was introduced into the mixture before resolving the emulsion by adding tetrahydrofuran. The resultant material was then separated and washed through centrifugation and dried overnight at 80 °C before being calcinated at 250 °C for 2.5 h. See Section S9 (Supporting Information) for additional characterizations.

### SFC‐ICP‐MS

The electrochemical online SFC‐ICP‐MS measurements were carried out employing an Ag/AgCl (sat. KCl, Metrohm) RE and a glassy carbon rod (HTW Sigradur G) CE. The RE is placed after the SFC outlet to avoid chloride contamination. A previously reported optimized version of the SFC‐ICP‐MS system was used to fulfil the strict cleanliness requirements when working with well‐defined surfaces.^[^ ^69^ ^]^ The working electrolyte was bubbled with Ar in a purge vial and was constantly fed using a peristaltic pump (Ismatec). The solution circulated through the SFC to the ICP‐MS (NexIONTM 350X, PerkinElmer) by matching the Ar flow in the purge vial and the rotation rate of the peristaltic pump (MP2, Elemental Scientific) of the ICP‐MS. A ca. 200 µL min^−1^ flow rate, carefully determined for each measurement, was employed. The dissolution of ^106^Pd from the working electrode was tracked with the ICP‐MS via previous calibration with Pd standard solutions (0, 0.5, 1, and 5 µg L^−1^, Certipur, Merck). ^103^Rh was the internal standard using a 10 µg L^−1^ Rh solution (Certipur, Merck). The working area of the electrode in contact with the solution was 0.159 cm^2^.

Two electrochemical protocols were applied using a Gamry Reference 600 potentiostat. The working electrode was always contacted to the electrolyte at a controlled potential of 0.4 V versus RHE in order to avoid any possible surface oxidation or hydrogen absorption before the measurement. The first protocol was a potential step program with sixty‐second‐long potential holds separated by 50 mV potential jumps, in which the initial potential value is 0.52 V and the final potential value is ‐0.18 V. The protocol was done backward up to 0.52 V, then repeated again to ‐0.18 V. The second protocol consisted of three consecutive CV cycles with a scan rate of 10 mV s^−1^ with 0.52 V and ‐0.18 V as UPL and LPL, respectively. All potentials in this work were converted to the RHE scale, and measurements were performed at room temperature (21 °C).

### MD Simulations

The large‐scale atomic/molecular massively parallel simulator (LAMMPS) package^[^ ^70^ ^]^ with the ML_PACE package^[^ ^25^ ^]^ was used for MD simulations in this study. The interatomic interactions were described using atomic cluster expansion (ACE)^[^ ^24^ ^]^ potentials, parametrized and iteratively trained for the Pd–H system based on a large, representative set of DFT calculations containing both elemental phases of Pd and H as well as binary structures of various compositions using the Pacemaker package.^[^ ^26^ ^]^ The ACE potential was validated thoroughly to ensure that it reproduces the fundamental properties of Pd and PdH crystals (see Section S10, Supporting Information). Details of the ACE model, its parametrization and computational benchmarks will be presented in a follow‐up study.

Since the primary objective of the simulations was not to replicate the full electrochemical mechanism of proton electro‐reduction, the MD simulations were carried out without applying electrostatic potential and without explicit liquid electrolyte. Typically, the complete process involves initial electrochemical hydrogen adsorption onto Pd surfaces (the Volmer reaction), followed by hydrogen diffusion into the subsurface which leads to the formation of a Pd hydride (PdH_x_) phase. Two distinct PdH*_x_* phases may (co)exist: the dilute solid‐solution phase called α‐PdH*_x_* (*x* < 0.03) and the metal hydride phase or β‐PdH*_x_* (*x* > 0.60), with a large lattice mismatch (3.5%) between them.^[^ ^71^, ^72^ ^]^ In this study, the focus was on exploring the dynamics of Pd surfaces upon hydride formation, specifically the strain relaxation mechanism at the interface between the α‐ and β‐PdH_x_ phases,^[^ ^73^ ^]^ and the subsequent surface restructuring and roughening. To achieve this, Pd slab models with hydrogen atoms pre‐inserted into the subsurface were employed, thus bypassing the initial hydrogen adsorption and diffusion steps. This approach allowed us to directly investigate surface reconstruction and roughening under conditions where the Pd surface already contains a high concentration of subsurface hydrogen (β‐PdH_x_). When β‐PdH_x_ dominates, strain accumulation occurs,^[^ ^22^, ^73^ ^]^ leading to surface roughness. Importantly, this roughness is induced by the strain relaxation of the hydride phase and is unrelated to the presence of a solvent. Accordingly, different Pd surfaces were modeled by cleaving the DFT‐optimized structure of a bulk fcc Pd unit cell with the lattice constant of 3.891 Å, in the Pd(111), Pd(110), and Pd(100) facets. The surfaces were built with 20 layers for the Pd(111) and Pd(100), containing 576 and 512 Pd atoms per layer, respectively, and 28 layers for the Pd(110), having 384 Pd atoms per layer. The three Pd surfaces were completely covered by one monolayer of H adatoms at the three‐fold fcc hollow site, the long bridge site, and the four‐fold hollow site for the Pd(111), Pd(110), and Pd(100) surfaces, respectively.^[^ ^41^ ^]^ The upper half of all studied surfaces were fully populated with subsurface hydrogen occupying the well‐defined octahedral interstitial sites.^[^ ^74^ ^]^ The resulting system sizes were Pd_11520_H_5760_ (*a* = 66.03 Å, *b* = 57.18 Å), Pd_10752_H_5376_ (*a* = 62.25 Å, *b* = 66.03 Å), and Pd_10240_H_5120_ (*a* = 62.25 Å, *b* = 62.25 Å) for the Pd(111), Pd(110), and Pd(100) surfaces, respectively.

Before performing the MD production runs, an energy minimization with constant cell shape and volume of all structures was employed with LAMMPS using the conjugate gradient method to obtain the stable structures, where the stopping tolerance was set to 10^−10^ eV normalized on the systems’ total energy and 10^−10^ eV Å^−1^ for the atomic forces. All MD simulations of the optimized structures were performed in the canonical (NVT) ensemble for 8 ns at room temperature (300 K) using the Nosé‐Hoover thermostat with a temperature damping parameter of 0.05 ps. The time step in all simulations was 1 fs. The final structures after 8 ns were minimized maintaining constant cell shape and volume via the conjugate gradient method for subsequent analysis. The Open Visualization Tool (OVITO) software^[^ ^75^ ^]^ was used for the visualization of surfaces, elastic strain calculation and dislocation structure analysis.

It is worth noting that the behavior of H adsorption and absorption in Pd surfaces is dependent on the Pd lattice constant. The previous work^[^ ^41^ ^]^ demonstrated that the DFT method using the Perdew, Burke, and Ernzerhof (PBE) functional, with the van der Waals (vdW)‐dispersion correction (PBE‐D3) proposed by Grimme et al.^[^ ^76^ ^]^ provides the lattice constant closer to the experimental value, compared to the PBE one. Therefore, in this work, the MD results were obtained by the ACE potential that was trained on the samples of PBE‐D3 calculations (ACE‐PBE‐D3 potential) in the main text. For comparison, MD simulations using the ACE potential without including the vdW correction (ACE‐PBE potential) were also performed on the PdH/Pd(111), PdH/Pd(110), and PdH/Pd(100) slab models, constructed from a bulk fcc Pd unit cell with the PBE‐lattice constant of 3.941 Å. These results, provided in Section S11 (Supporting Information), are qualitatively similar to those presented in the main text.

## Conflict of Interest

The authors declare no conflict of interest.

## Supporting information



Supporting Information

## Data Availability

The data that support the findings of this study are available from the corresponding author upon reasonable request.
