# Supplementary material for: Hydride‐Induced Reconstruction of Pd Electrode Surfaces: A Combined Computational and Experimental Study
Source: Adv Mater. 2024 Dec 4;37(4):2410951. doi: 10.1002/adma.202410951 (PMC11775870; doi:10.1002/adma.202410951)
Supplement: Supplementary file 1 — Supporting Information [file ADMA-37-2410951-s001.pdf]

ADVANCED MATERIALS

Supporting Information

for *Adv. Mater.*, DOI 10.1002/adma.202410951

Hydride-Induced Reconstruction of Pd Electrode Surfaces: A Combined Computational and Experimental Study

Apinya Ngoipala, Christian Schott, Valentin Briega-Martos, Minaam Qamar, Matous Mrovec, Sousa Javan Nikkhah, Thorsten O. Schmidt, Lewin Deville, Andrea Capogrosso, Lilian Moumaneix, Tanja Kallio, Arnaud Viola, Frédéric Maillard, Ralf Drautz, Aliaksandr S. Bandarenka, Serhiy Cherevko, Matthias Vandichel* and Elena L. Gubanova**

Supporting Information

Hydride-Induced Reconstruction of Pd Electrode Surfaces: A Combined Computational and Experimental Study

Apinya Ngoipala,[1,#] Christian Schott,[2,#] Valentin Briega-Martos,[3,#] Minaam Qamar,[4,#] Matous Mrovec,[4] Sousa Javan Nikkhah,[1] Thorsten O. Schmidt,[2] Lewin Deville,[2] Andrea Capogrosso,[2] Lilian Moumaneix,[5] Tanja Kallio,[5] Arnaud Viola,[6] Frédéric Maillard,[6] Ralf Drautz,[4] Aliaksandr S. Bandarenka,[2,7] Serhiy Cherevko,[3,] Matthias Vandichel,[1,*] Elena L. Gubanova[2,*]*

[1] School of Chemical Sciences and Chemical Engineering, Bernal Institute, University of Limerick, Limerick, V94 T9PX Ireland

[2] Physics of Energy Conversion and Storage, Department of Physics, Technical University of Munich, James-Franck-Straße 1, 85748, Garching, Germany.

[3] Helmholtz Institute Erlangen-Nürnberg for Renewable Energy (IET-2), Forschungszentrum Jülich GmbH, Cauerstr. 1, 91058 Erlangen, Germany

[4] Interdisciplinary Centre for Advanced Materials Simulation (ICAMS), Ruhr-Universität Bochum, 44780 Bochum, Germany

[5] Department of Chemistry and Materials Science, Aalto University, Kemistintie 1, Espoo, 02150, Finland

[6] Université Grenoble Alpes, Université Savoie Mont Blanc, CNRS, Grenoble INP, LEPMI, Grenoble, 38000 France

[7] Catalysis Research Center TUM, Ernst-Otto-Fischer-Str. 1, 85748, Garching, Germany

[#] These authors contributed equally to this work

[*] Corresponding authors:

s.cherevko@fz-juelich.de (S. Cherevko)

matthias.vandichel@ul.ie (M. Vandichel)

elena.gubanova@tum.de (E. L. Gubanova)

Section S1: Investigating morphological changes on Pd basal planes after potential cycling

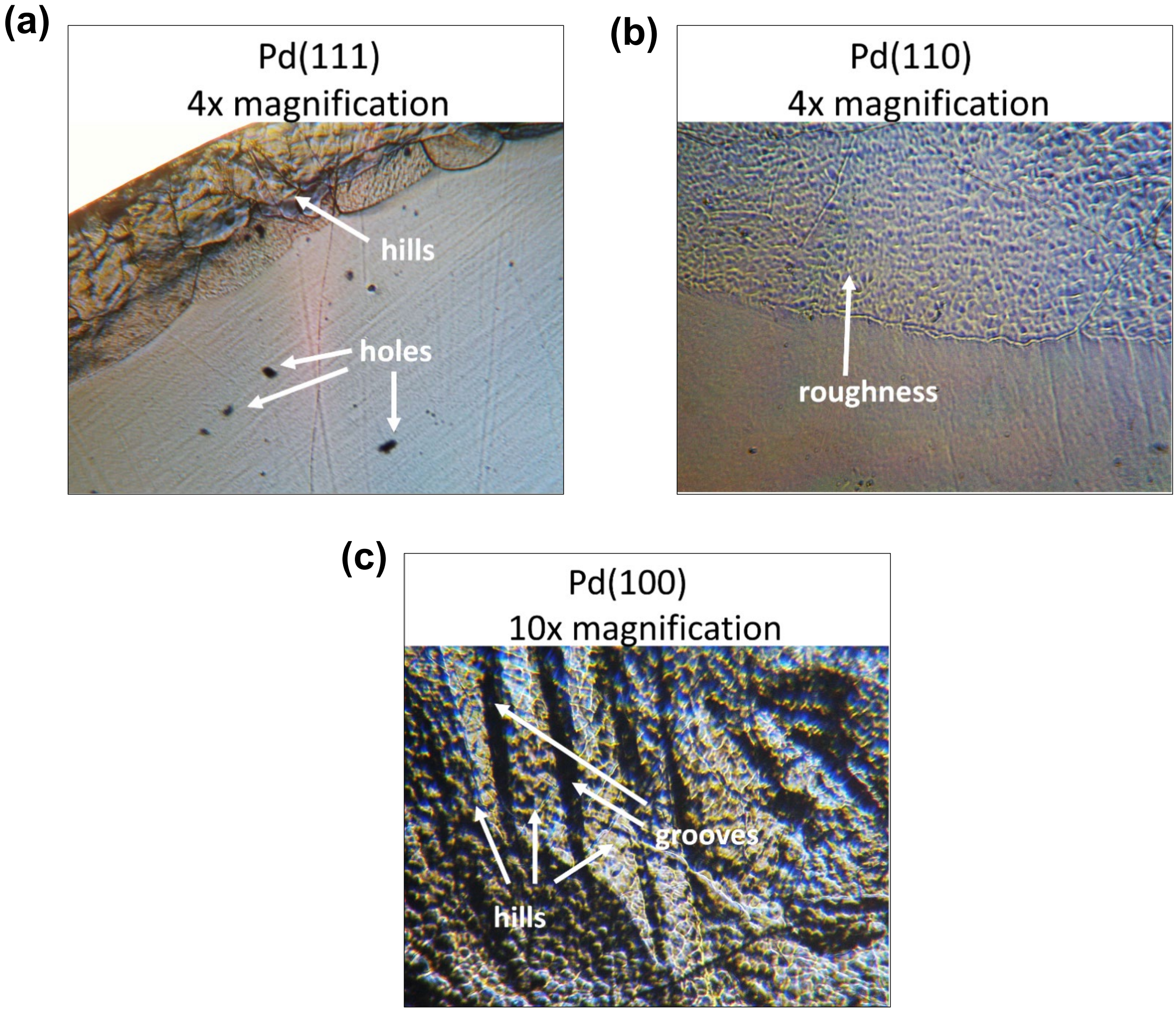

Figure S1. Optical microscopy images of a) Pd(111), b) Pd(110), and c) Pd(100) basal single crystal surfaces after potential cycling in 0.1 M $HClO_4$ with 4x, 4x, and 10x magnification, respectively. Morphological surface changes are visible as hills, grooves, and a general increase in roughness.

Table S1. EC-STM measurement parameters for all images obtained related to the respective Pd single crystal in **Figures 2**b-d and **3**a,b.

single crystal	scan size / nm	current setpoint / nA	scan rate / Hz	E_{work} / mV vs. RHE	E_{tip} / mV vs. RHE	integral gain	proportional gain
Pd(111)	150	3.0	1.6	531	501	0.3	0.6
Pd(100)	200	3.0	1.25	581	532	0.6	1.0
Pd(110)	200	4.5	1.25	581	531	0.6	1.0

Table S2. Parameters for conducted CVs of the basal Pd single crystals recorded within the EC-STM cell for the investigations of morphological surface changes. A summary of recorded CVs and EC-STM images is shown in **Figures S2-S4**.

single crystal	cycle numbers	E_{min} / mV vs. RHE	E_{max} / mV vs. RHE
Pd(111)	1-44	180	530
Pd(100)	1-30	64	564
Pd(110)	1-6	124	564
Pd(110)	7-28	144	564

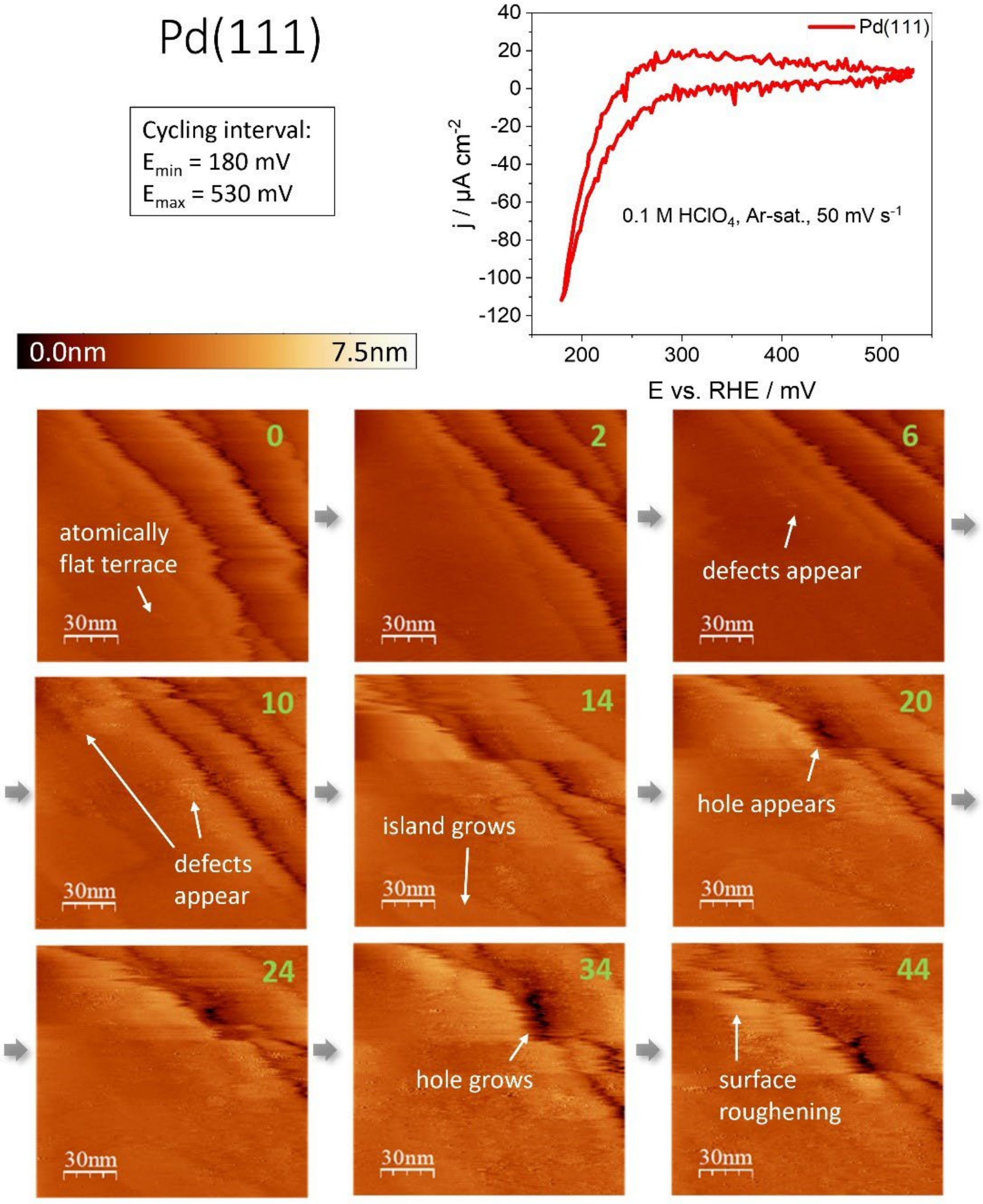

Figure S2. Summary of all recorded surface snapshots on (111) basal Pd single crystals using EC-STM methods. The used potential ranges and representative CV cycles are displayed at the top of the figure. The number of CV cycles completed before each EC-STM image was taken is indicated in green at the top right corner of each EC-STM image.

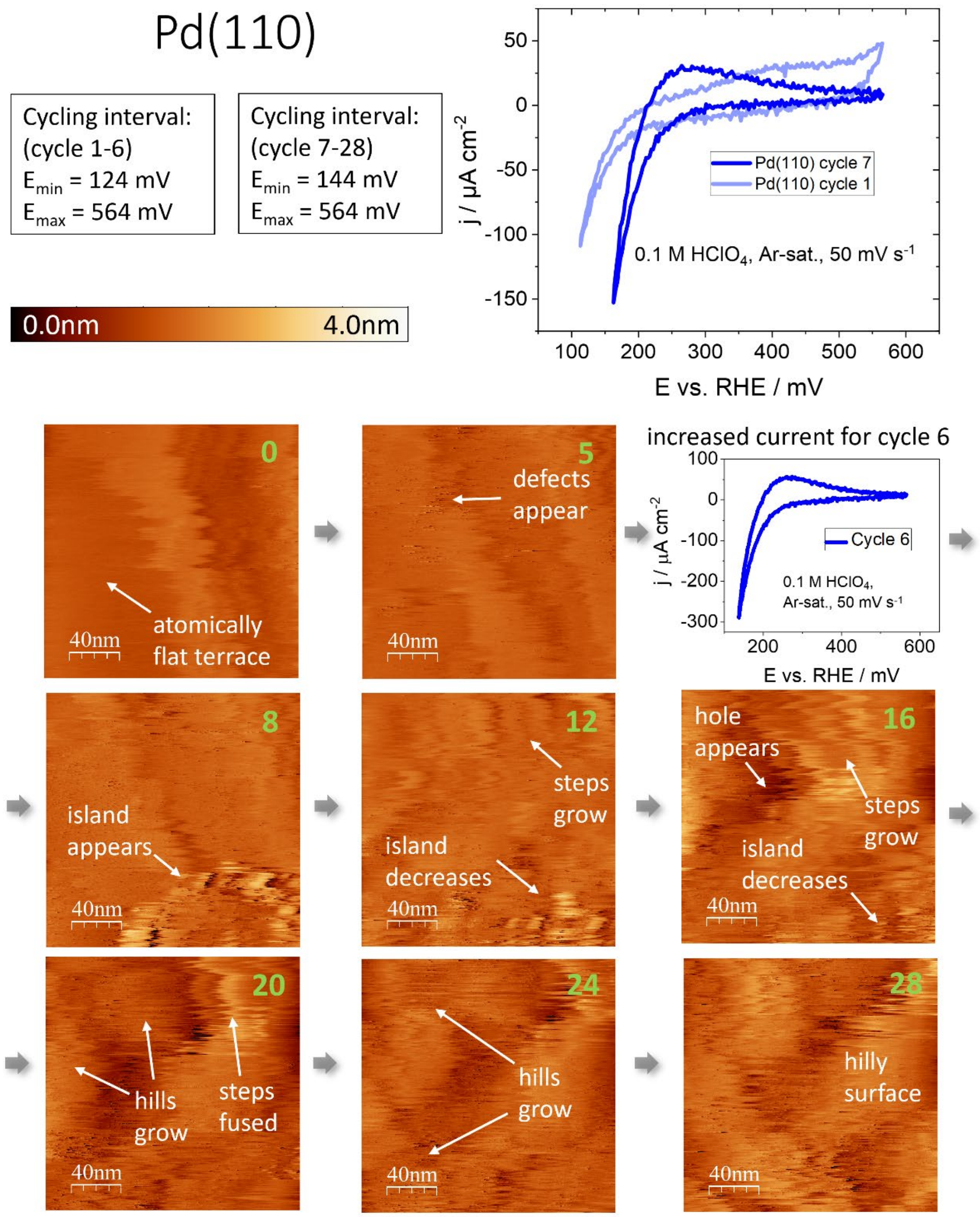

Figure S3. Summary of all recorded surface snapshots on (110) basal Pd single crystals using EC-STM methods. The used potential ranges and representative CV cycles are displayed at the top of the figure. The number of CV cycles completed before each EC-STM image was taken is indicated in green at the top right corner of each image. Due to a significant increase in current density during the sixth potential cycle, the potential range was subsequently adjusted (highlighted by the dark blue CV) to achieve roughly consistent current densities.

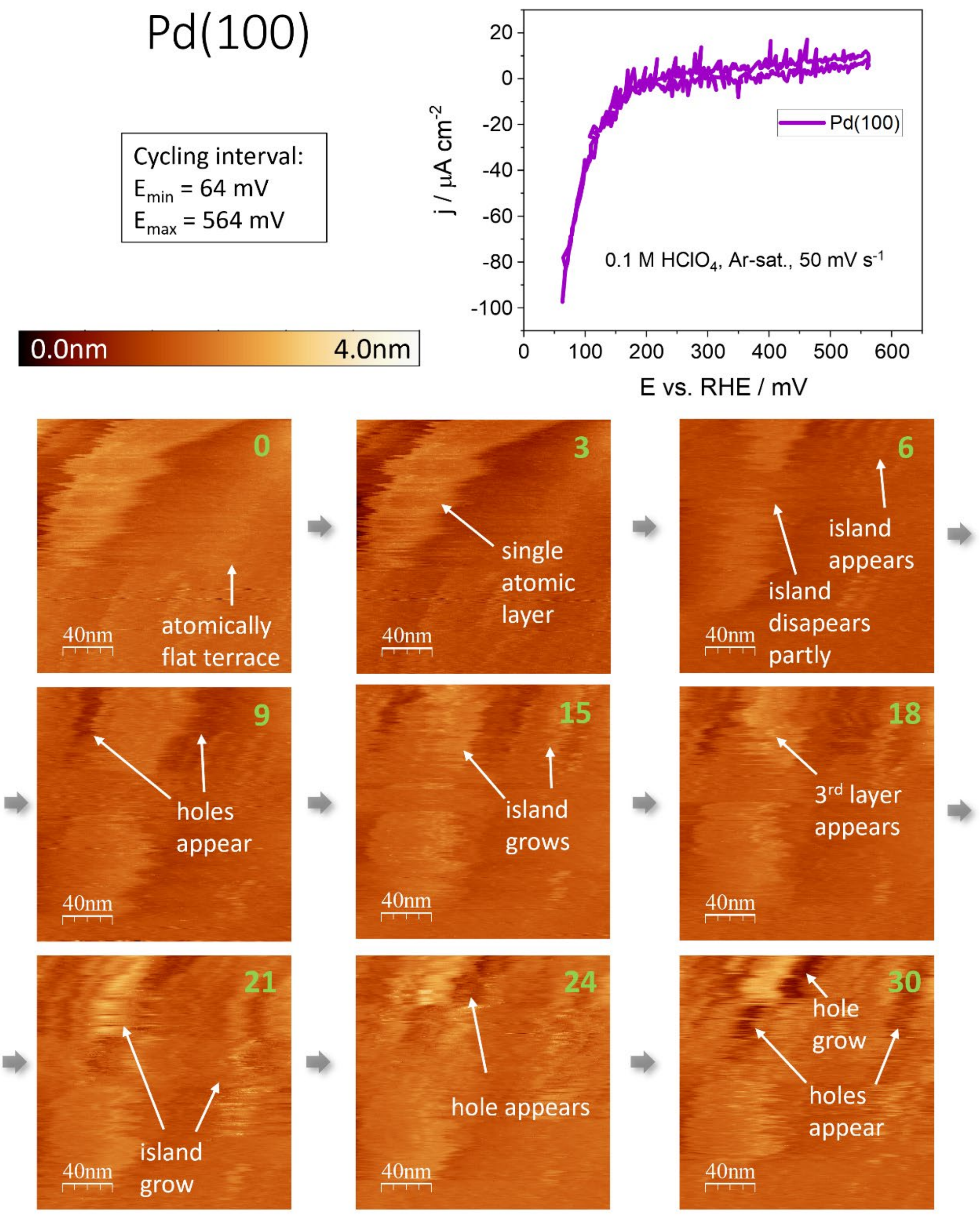

Figure S4. Summary of all recorded surface snapshots on (100) basal Pd single crystals using EC-STM methods. The used potential ranges and representative CVs cycles are displayed at the top of the figure. The number of CV cycles completed before each EC-STM image was taken is indicated in green at the top right corner of each image.

Roughness Analysis of EC-STM images

Classical RMS roughness analysis relies on the distance of each individual point relative to the average surface profile.[1] Consequently, nanometer-sized features, such as hills and valleys, significantly influence the RMS value, whereas sub-nanometer features, like spikes, have a lesser impact. To accentuate the surface roughness at sub-nanometer scales, we utilize a modified version of the standard RMS tool that focuses on surface derivatives instead of actual surface height values. These derivatives are estimated by calculating the height difference between two adjacent measurement points. This is illustrated in **Equation 1**, where the height of the previous point z_{-1} is subtracted from the current point z, with both points defined by their respective lateral coordinates x and y. These values are subsequently squared, and the calculation is repeated for each measurement point to determine the average. The square root of this average yields an RMS value that captures surface roughness while emphasizing the influence of sub-nanometer features.

This modified RMS analysis is applied to selected EC-STM images recorded after a specific CV cycle number. The resulting data is presented in **Figure 3**d, highlighting a clear increase in roughness with subsequently conducted CV cycles.

$$R_{\mathrm{RMS}} = \sqrt{\frac{1}{A\,B} \sum_{a=1}^{A} \sum_{b=1}^{B} [z\,(x_a, y_b) - z_{-1}\,(x_a, y_b)\,]^2} \quad (1)$$

Section S2: On-line dissolution measurements

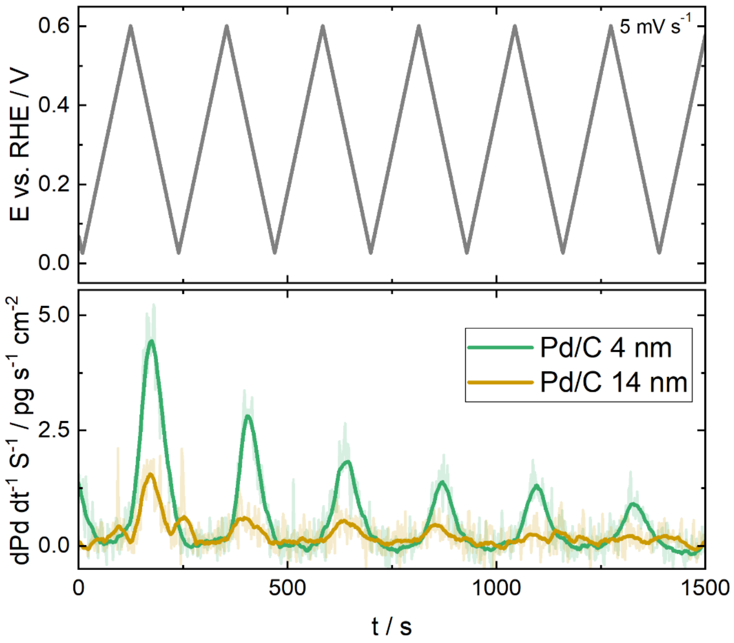

Figure S5. Dissolution profiles for Pd/C materials with an average particle size of 4 nm and 14 nm in Ar-saturated 0.1 M $HClO_4$. The electrochemical protocol consisted of successive cyclic voltammetries from 0.03 V to 0.60 V vs. RHE at a scan rate of 5 mV s^{-1}, in accordance with the previous work by Viola et al.[2]

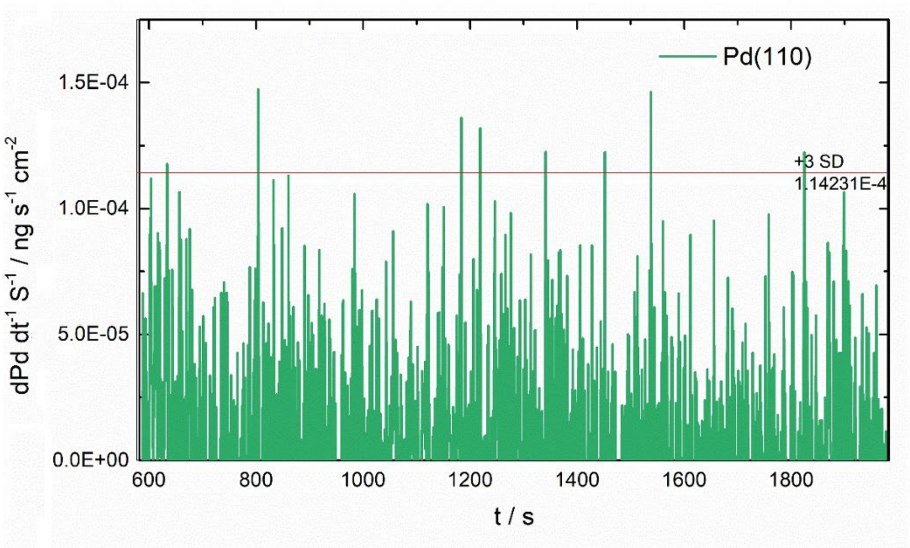

Figure S6. Example for the visualization of the detection limit for the on-line dissolution measurements. The detection limit can be determined as the value for which the signal-to-noise ratio is three (S/N = 3). Defining the noise as the standard deviation of a signal-free portion of the data, then the detection limit corresponds to three times that standard deviation.

Section S3: MD simulations of the PdH/Pd(hkl) systems using the ACE-PBE-D3 potential

MD simulations were performed on the Pd(111), Pd(110), and Pd(100) slab models containing both surface and subsurface hydrogen (see **Section 4**, **Methods – MD simulations** in the main text). An energy minimization was first employed to obtain the stable initial structures as depicted in **Figure S7**. Subsequently, MD annealing for 8 ns at 300 K using the NVT ensemble was conducted for all three minimized surfaces and their surface formation energies ($\gamma_{\mathrm{PdH/Pd,slab}}$) were calculated as:

$$\gamma_{\mathrm{PdH/Pd,slab}} = \frac{1}{2A}\left(E_{\mathrm{PdH/Pd,slab}} - N\mathrm{E}_{\mathrm{Pd_1H_{0.5},bulk}}\right), \quad (2)$$

where A is the surface area of the slab; $E_{\mathrm{PdH/Pd,slab}}$ and $\mathrm{E}_{\mathrm{Pd_1H_{0.5},bulk}}$ are the total energy of the PdH/Pd(hkl) surface slab and the total energy of the bulk system with the H/Pd ratio of 0.5, respectively; N is the number of atoms in the slab divided by the number of atoms in the bulk system. Here, the total energy of the bulk $Pd_1H_{0.5}$ system was obtained by MD simulation using NPT ensemble for 6 ns at 300 K using the Nosé-Hoover thermostat and barostat where the simulation conditions were set in a similar fashion as for the MD NVT simulations of the PdH/Pd(hkl) slab models (see **Section 4**, **Methods – MD simulations** in the main text), and calculating the average total energy over the equilibrium period. Note that an 8×8×8 supercell of the bulk PdH unit cell with the lattice constant of 4.079 Å was constructed to obtain the bulk $Pd_1H_{0.5}$ system containing 2048 Pd atoms and 1024 H atoms. The calculated surface formation energies as a function of time were plotted for all three surfaces, as shown in **Figure S10**. The PdH/Pd(111) and PdH/Pd(100) surfaces appear relatively stable, with the surface energy reaching a plateau after about 5 ns of simulations, whereas the surface energy of PdH/Pd(110) gradually decreases over time. Based on the calculated surface formation energies, the Pd(111) case is the most stable surface, followed by the (100) and (110) surfaces, in good agreement with the trend of increasing surface energies of H-covered Pd, [(110) < (100) < (111)] as reported in the previous DFT study.[3]

To quantify the surface roughness, the root mean square (RMS) roughness value was calculated as:

$$R_{\mathrm{RMS}} = \sqrt{\frac{\sum_{i=1}^{n}(z_i-\bar{z})^2}{n}}, \quad (3)$$

where z_i, $\bar{z}$, and n are the height of the exposed surface atom i, the mean height of the exposed surface atoms, and the total number of exposed surface atoms considered, respectively.[1] The calculated RMS roughness of the three Pd surfaces as a function of time is illustrated in **Figure 5**d.

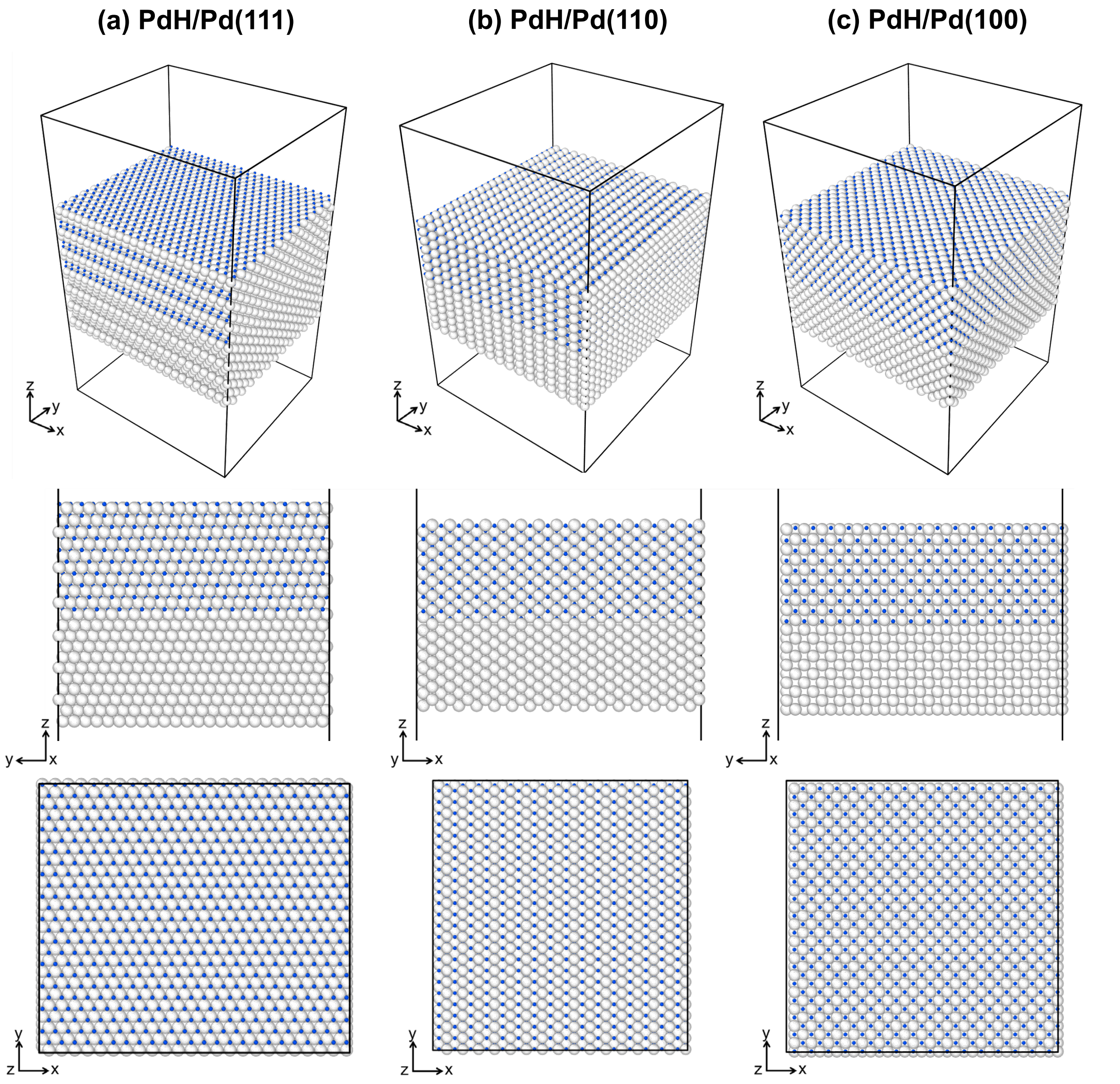

Figure S7. Optimized initial structures of a) PdH/Pd(111), b) PdH/Pd(110), and c) PdH/Pd(100) shown in different views, where the white and blue balls represent Pd and H atoms, respectively.

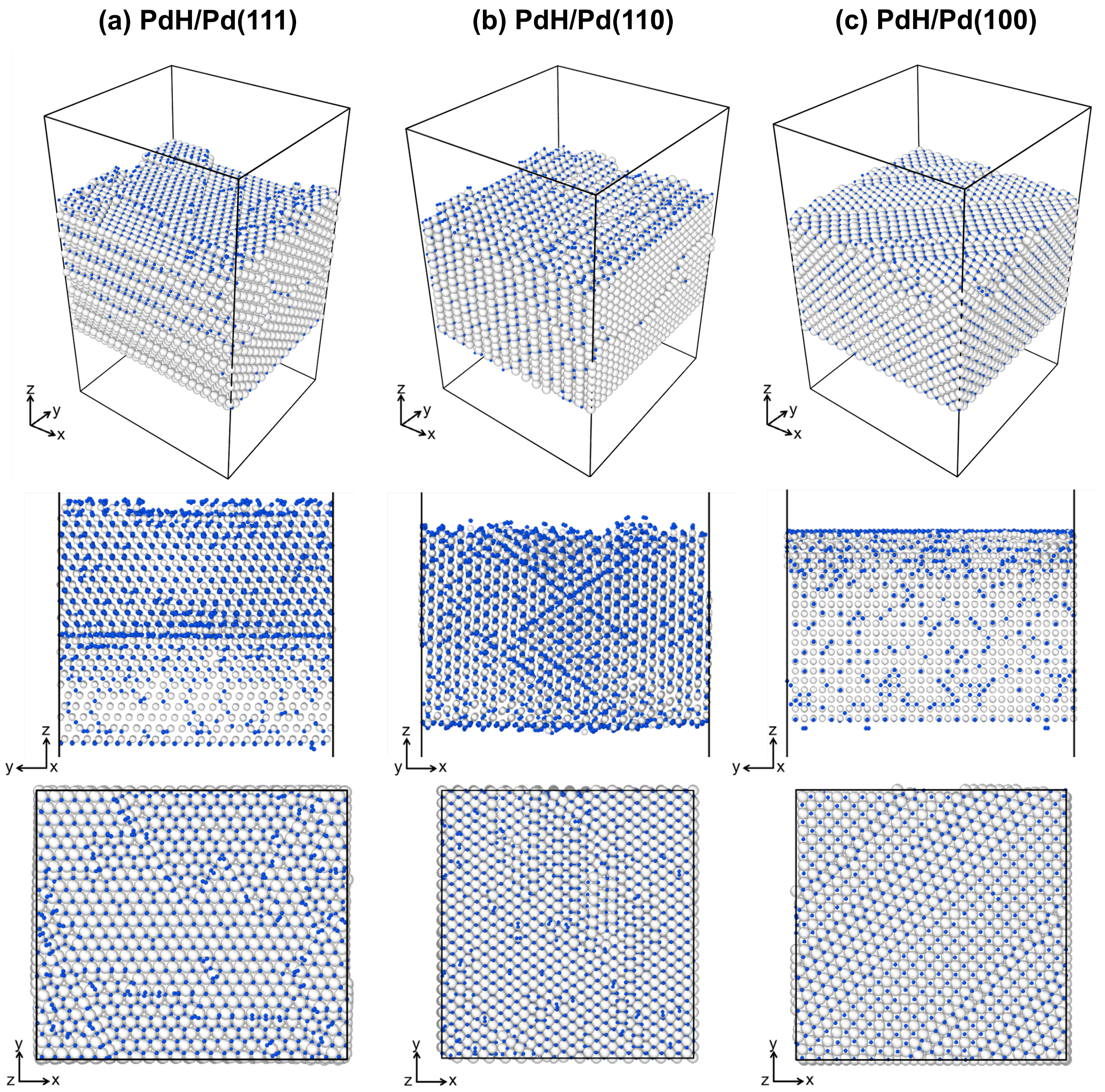

Figure S8. Reoptimized structures after 8 ns MD equilibration at 300 K of a) PdH/Pd(111), b) PdH/Pd(110), and c) PdH/Pd(100) shown in different views, where the white and blue balls represent Pd and H atoms, respectively.

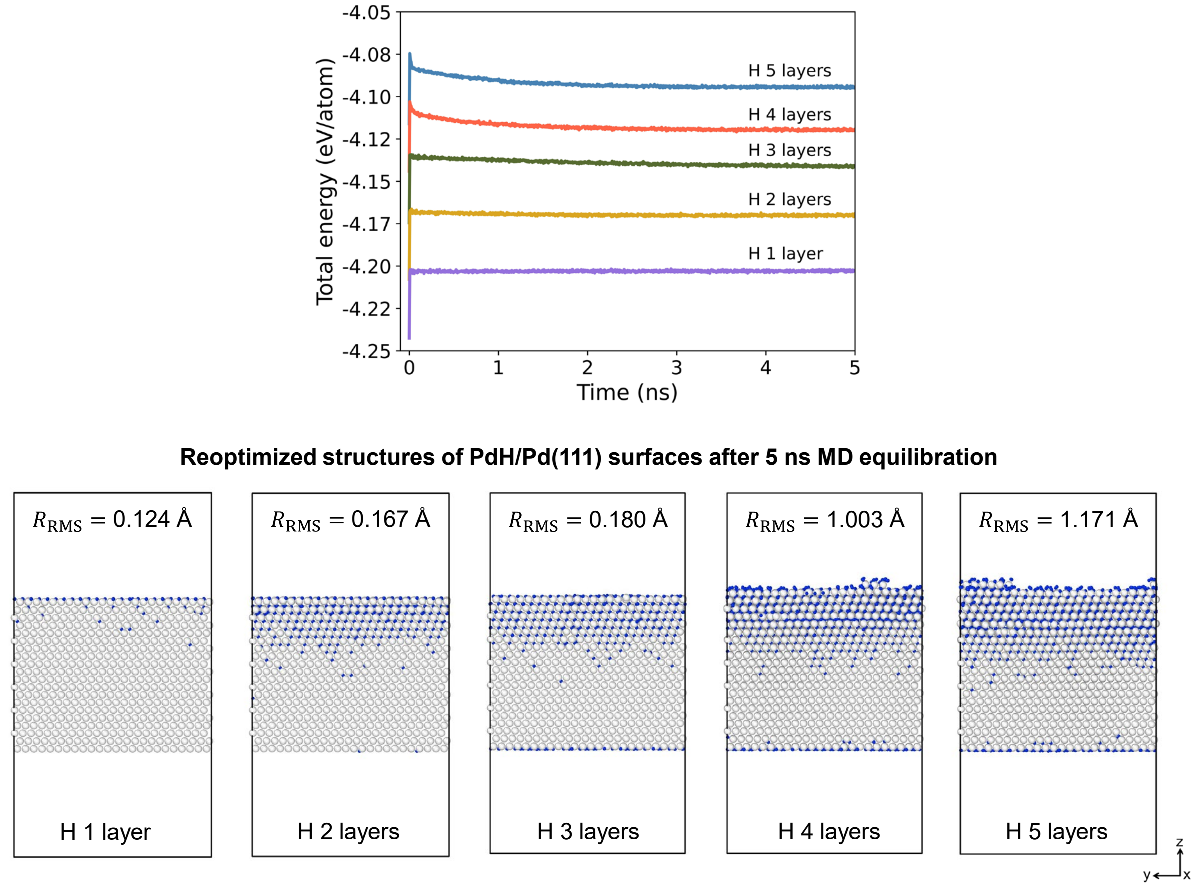

Figure S9. Total energy as a function of time during MD simulations (0-5 ns) at 300 K for PdH/Pd(111) surfaces with varying number of hydrogen layers (top panel) and their corresponding reoptimized structures after 5 ns MD equilibration with the calculated root mean square roughness (R_{RMS}) values (bottom panel).

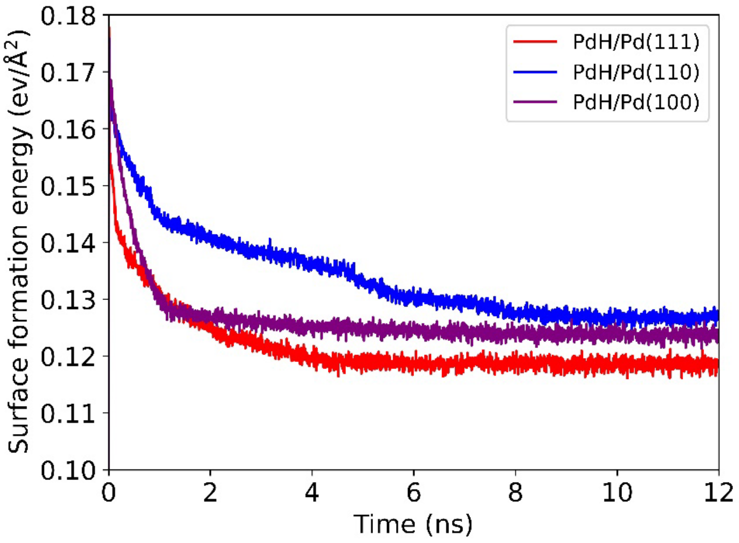

Figure S10. Calculated surface formation energy as a function of time during MD simulations at 300 K of PdH/Pd(111), PdH/Pd(110), and PdH/Pd(100). This confirms that the surfaces were already equilibrated. In other words, the presented surfaces were based on equilibrated simulations.

Section S4: H adsorption configurations on the surfaces of the equilibrated PdH/Pd(hkl) systems

On the PdH/Pd(111) surface, from the beginning, all H atoms stay at the threefold fcc hollow site (see **Figure 7**a). After 8 ns MD equilibration at 300 K (see **Figure S11**a), H mostly resides at the fcc hollow site on the two step layers, while the bridge sites are populated by H at the step edges. Notably, all H atoms occupy the hcp hollow site on the remaining exposed surface due to the repulsive interaction between the adsorbed H on the surface and the absorbed H in the first-sublayer octahedral site. Since the octahedral site is located directly underneath the fcc hollow site on the surface (the most favorable surface site for H adsorption), the H–H distance between these two sites is insufficient to minimize their repulsive interaction. Consequently, H on the surface moves to a position farther away from the subsurface H, *i.e.*, the hcp hollow site. This finding is consistent with the previous DFT study.[3] For the PdH/Pd(110) case, initially, all H atoms stay at the long bridge sites (see **Figure S7**b), however, when the reconstruction happens during the first 8 ns of the MD simulation, H atoms occupy the pseudo threefold, long bridge, and short bridge sites (see **Figure S11**b). In case of the PdH/Pd(100) surface, all H atoms adsorb on the surface at the fourfold hollow sites for the initial structure before MD (see **Figure S7**c). For the structure after 8 ns MD equilibration, due to the emergence of (111) facet, surface H occupies the threefold fcc hollow site on the (111) orientation, and most H atoms reside at the

fourfold hollow site on the (100) orientation while the bridge site is occupied by one H atom (see **Figure S11**c).

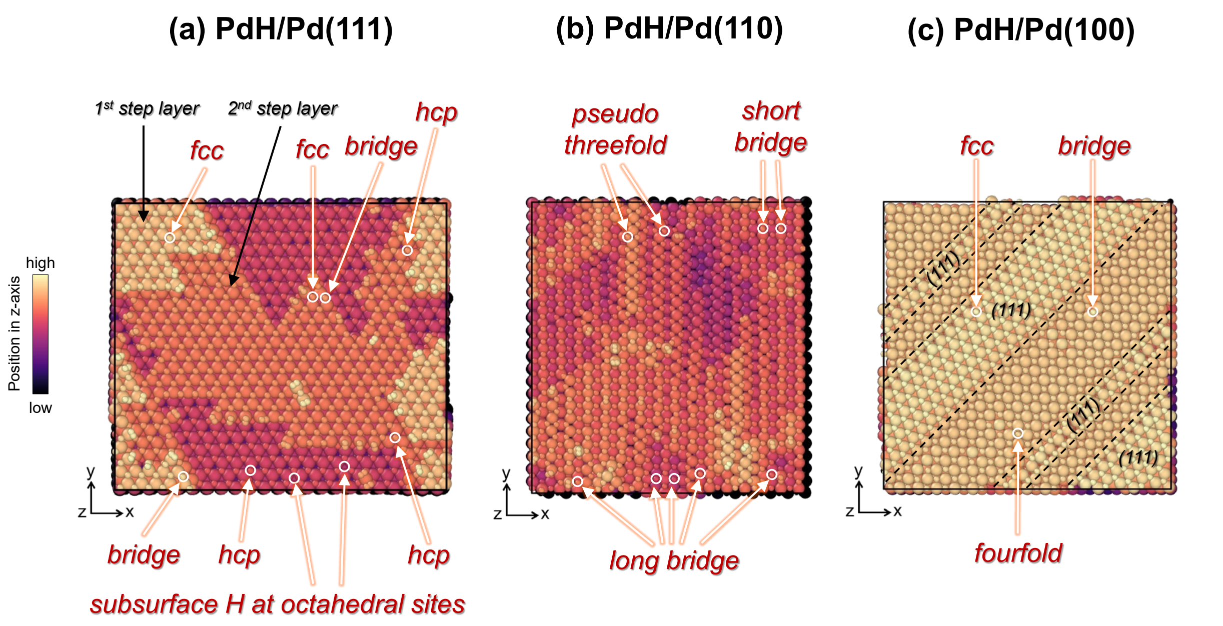

Figure S11. Reoptimized structures after 8 ns MD equilibration at 300 K of a) PdH/Pd(111), b) PdH/Pd(110), and c) PdH/Pd(100) shown in top view, with color gradient indicating the position of all atoms along the *z* direction, where different adsorption sites for H are illustrated.

Section S5: Nearest-neighbor analysis for the equilibrated PdH/Pd(hkl) systems

We conducted a nearest-neighbor analysis to investigate the local atomic environment of H atoms with respect to Pd atoms within the crystal lattice. The nearest neighbor analysis conducted in this study offers valuable information regarding the spatial arrangement of Pd and H atoms. Here, we first calculated the radial distribution function $g_{ij}(r)$, which is determined by analysing a distance histogram constructed by counting the number of atoms located in spherical shells of radius r and thickness:

$$g_{ij}(r) = (4\pi r^2 \rho\, \Delta r)^{-1} < N_{ij}(r; \Delta r) >, \tag{4}$$

where, ρ represents the average number density, Δr is the bin width of the distance histogram, N_{ij} is the number of i sites surrounding j sites within a distance ranging from $r-\Delta r/2$ to $r+\Delta r/2$, and the angle brackets indicate averaging over trajectory data collected during the last 3 ns of MD simulations. The g(r) results between Pd and H for the surface and bulk systems are presented in **Figure S12**. In the simulations employing the ACE-PBE-D3 potential, the first peak of the radial distribution function between Pd and H atoms is slightly shifted to a greater distance than in systems simulated with the ACE-PBE potential. This observation can be attributed to the abundance of H atoms occupying tetrahedral sites within the ACE-PBE-simulated systems. However, in the bulk system, the first peak in the radial distribution function graph for the system using the ACE-PBE potential appears at a slightly greater distance compared to the system simulated with the ACE-PBE-D3 potential.

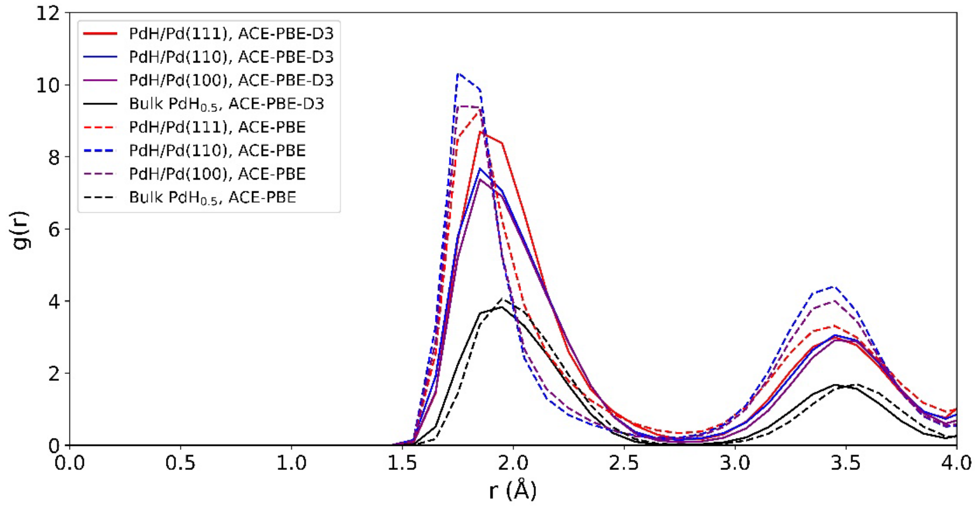

Figure S12. Average radial distribution functions between Pd and H atoms for the PdH/Pd(111), PdH/Pd(110), PdH/Pd(100), and bulk $PdH_{0.5}$ systems obtained from MD simulations using ACE-PBE-D3 and ACE-PBE potentials.

Next, the distances between each H atom and all Pd atoms (including the periodic boundary condition effect) were calculated to assess the coordination of H atoms with neighboring Pd atoms. The cutoff distance used for these calculations is 2.4 Å, determined from the radial distribution function results (see **Figure S12**). The number of Pd atoms within this cutoff distance around each H atom was then determined. The distribution of nearest Pd neighbors for the H atoms in the equilibrated PdH/Pd(111), PdH/Pd(110), PdH/Pd(100), and bulk $PdH_{0.5}$ systems is presented in **Figure 5**e. It is obvious from **Figure 5**e that most of the absorbed hydrogens reside in octahedral interstitial sites (6 Pd nearest neighbors) for all studied surfaces. All systems also contain hydrogen in tetrahedral sites (4 Pd nearest neighbors) and hydrogen with pyramidal Pd surroundings (5 Pd nearest neighbors) due to the dynamics and distortion in the systems. The greatest number of H atoms surrounded by 3 Pd atoms was found in the (111) case, followed by the (110), and (100) systems. These findings indicate the favorable H adsorption sites, *i.e.*, 3-fold fcc hollow site on the Pd(111) surface and pseudo 3-fold site on the Pd(110) surface; and the 3-fold fcc hollow site on the (111) orientation occurring at the surface of Pd(100). Moreover, H atoms are surrounded by one and two Pd atoms, implying the occupied top and bridge sites on the surfaces, respectively, which were found mostly in the (111), and (110) systems.

Furthermore, we explored the spatial relationships between H and Pd atoms by plotting H atoms and assigning colors based on their Pd nearest neighbor counts (see **Figures S17-S19** and **S23** for results obtained from ACE-PBE-D3 and ACE-PBE potentials, respectively). This visualization technique allows for a direct representation of the local atomic environment surrounding each atom type. It provides a clear illustration of the distribution of coordination environments within the crystal lattice, highlighting regions with differing levels of coordination and shedding light on the atomic-scale structure-property relationships within the material.

Section S6: Distributions of Pd and H atoms and H content in the equilibrated PdH/Pd(hkl) systems

To estimate the H content on Pd layers, we quantify the distribution of Pd and H atoms within the PdH/Pd(111), PdH/Pd(110), PdH/Pd(100) systems. Utilizing the k-means clustering algorithm,[4] we partitioned the Pd atoms into distinct layers within the lattice. The k-means clustering partitions a dataset into a predefined number of clusters by iteratively updating centroids based on data point assignments. It assigns each point to the nearest centroid and updates centroids. This process repeats until convergence. The output includes cluster assignments and final centroids, offering a concise and efficient way to analyse data structure. Following the clustering analysis, we determined bin edges based on the cluster centres obtained from the Pd layers. These bin edges served to delineate the boundaries of spatial layers within the Pd lattice.

With the bin edges established, we computed histograms to quantify the number of H atoms falling within each spatial bin. This enables us to assess the distribution and density of H atoms within the Pd planes. By analysing the histogram data, we estimated the H content on each Pd layer. Through H content analysis, we were able to assess the spatial distribution of H atoms and estimated H content on Pd layers. This information is crucial for understanding the interaction between H and Pd in the system.

The resulting distributions of Pd and H atoms along *z* direction in the equilibrated PdH/Pd(111), PdH/Pd(110), PdH/Pd(100) systems are presented in **Figure 5**f-h. With this analysis, the H content on each Pd layer were obtained as illustrated in **Figure S13**. Note that here, the H content is defined as the ratio of adsorbed/absorbed H atoms to Pd atoms in the considered layer, in units of monolayer (ML). For the Pd(111) case (**Figure S13**a), 1.73 ML and 0.89 ML H contents were found on the 1st and 2nd step layers, respectively, whereas the next 11 layers hold about 0.57-0.77 ML of H. The H content decreases in the following Pd layers, in which the last 6 sublayers accommodate less hydrogen with only about 0.02-0.06 ML. The H content reaches 0.98 ML on the other surface side (last layer). Similarly, for the Pd(110) case (**Figure S13**b), the Pd adlayer together with adrows are covered by H with 2.66 ML and the next 15 layers have the H contents in the range between 0.94 ML and 0.50 ML. A lower number of H are absorbed in the subsequent layers, varying from 0.49 ML to 0.12 ML, where 1.38 ML H content is found on another surface side (last layer). For the case of (100) termination (**Figure S13**c), the configuration seems to be more equilibrated among other two surfaces since the number of Pd atoms in each layer remains the same as in the initial structure, resulting in 1.02 ML and 1.01 ML H contents on the two surface sides, and the contents in the range between

0.21 ML and 0.62 ML for the sublayers. Note that the high H content on the surface includes some H_2 formation (see for example **Figures S17-S19**).

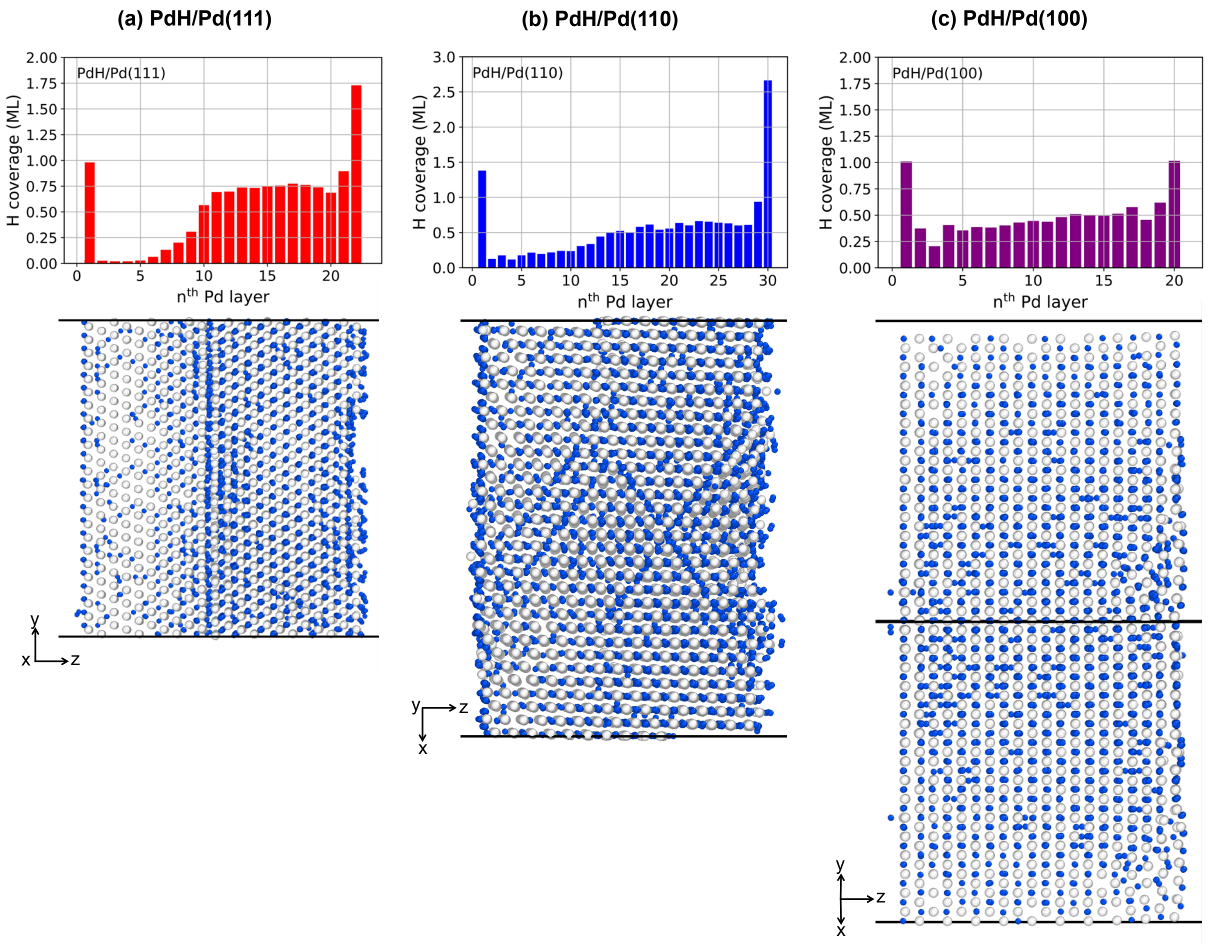

Figure S13. H content (in units of monolayer (ML)) on each Pd layer in the reoptimized structures after 8 ns MD equilibration at 300 K using the ACE-PBE-D3 potential for a) PdH/Pd(111), b) PdH/Pd(110), and c) PdH/Pd(100). Visualization of these model systems in *z* direction is also presented in the lower panel, where the white and blue balls represent Pd and H atoms, respectively.

Section S7: H Diffusion in the PdH/Pd(hkl) systems

To comprehensively understand the dynamics of the PdH/Pd(111), PdH/Pd(110), and PdH/Pd(100) systems, we calculated the mean square displacement (MSD) in all six spatial dimensions: xx, yy, zz, xy, yz, and xz. This analysis provides insights into the directional diffusion behaviour of H atoms within the Pd lattice. In anisotropic systems, diffusion may depend on the direction and, in general, the MSD is obtained as a tensor:

$$\boldsymbol{MSD} = \langle[\boldsymbol{r}(t+\Delta t) - \boldsymbol{r}(t)] \otimes [\boldsymbol{r}(t+\Delta t) - \boldsymbol{r}(t)]\rangle, \quad (5)$$

where $\otimes$ is a $\boldsymbol{r}$ tensor product. The tensor is symmetric with 6 unique elements: xx, yy, zz, $xy = yx$, $yz = zy$, and $xz = zx$; given by

$$MSD_{xx} = \langle[x(t+\Delta t) - x(t)]^2\rangle, \quad (6)$$

$$MSD_{yy} = \langle[y(t+\Delta t) - y(t)]^2\rangle, \quad (7)$$

$$MSD_{zz} = \langle[z(t+\Delta t) - z(t)]^2\rangle, \quad (8)$$

$$MSD_{xy} = \langle[x(t+\Delta t) - x(t)][y(t+\Delta t) - y(t)]\rangle, \quad (9)$$

$$MSD_{yz} = \langle[y(t+\Delta t) - y(t)][z(t+\Delta t) - z(t)]\rangle, \quad (10)$$

$$MSD_{xz} = \langle[x(t+\Delta t) - x(t)][z(t+\Delta t) - z(t)]\rangle. \quad (11)$$

The MSD results of H atoms in the PdH/Pd(111), PdH/Pd(110), and PdH/Pd(100) systems are presented in **Figure S14**a-c, where the MSD of bulk $PdH_{0.5}$ are also calculated and shown in **Figure S14**d for comparison.

To extract the diffusion coefficient from MSD data, we applied Einstein-Smoluchowski relation[5-6], which relates the diffusion coefficient (D) to the MSD. The equation can be generalized to relate the MSD tensor and the diffusion tensor as follows:

$$\boldsymbol{D} = \frac{1}{2d}\frac{\boldsymbol{MSD}\,(\Delta t)}{\Delta t}, \quad (12)$$

where d is the dimensionality of the system, and Δt is the time interval. By examining the MSD plots, we computed the diffusion coefficients along different directions within a two-dimensional space (d = 2) for planar diffusion and a one-dimensional space (d = *1*) for axial diffusion). This involved fitting a linear model of the MSD data with respect to the lag-time. Subsequently, the fitting procedure yielded the diffusion tensor, which characterizes the anisotropic diffusion behaviour of H atoms within the Pd lattice. The components of this tensor denote the diffusion coefficients along the principal axes and their interrelations, providing valuable insights into the directional dynamics of H diffusion within the systems.

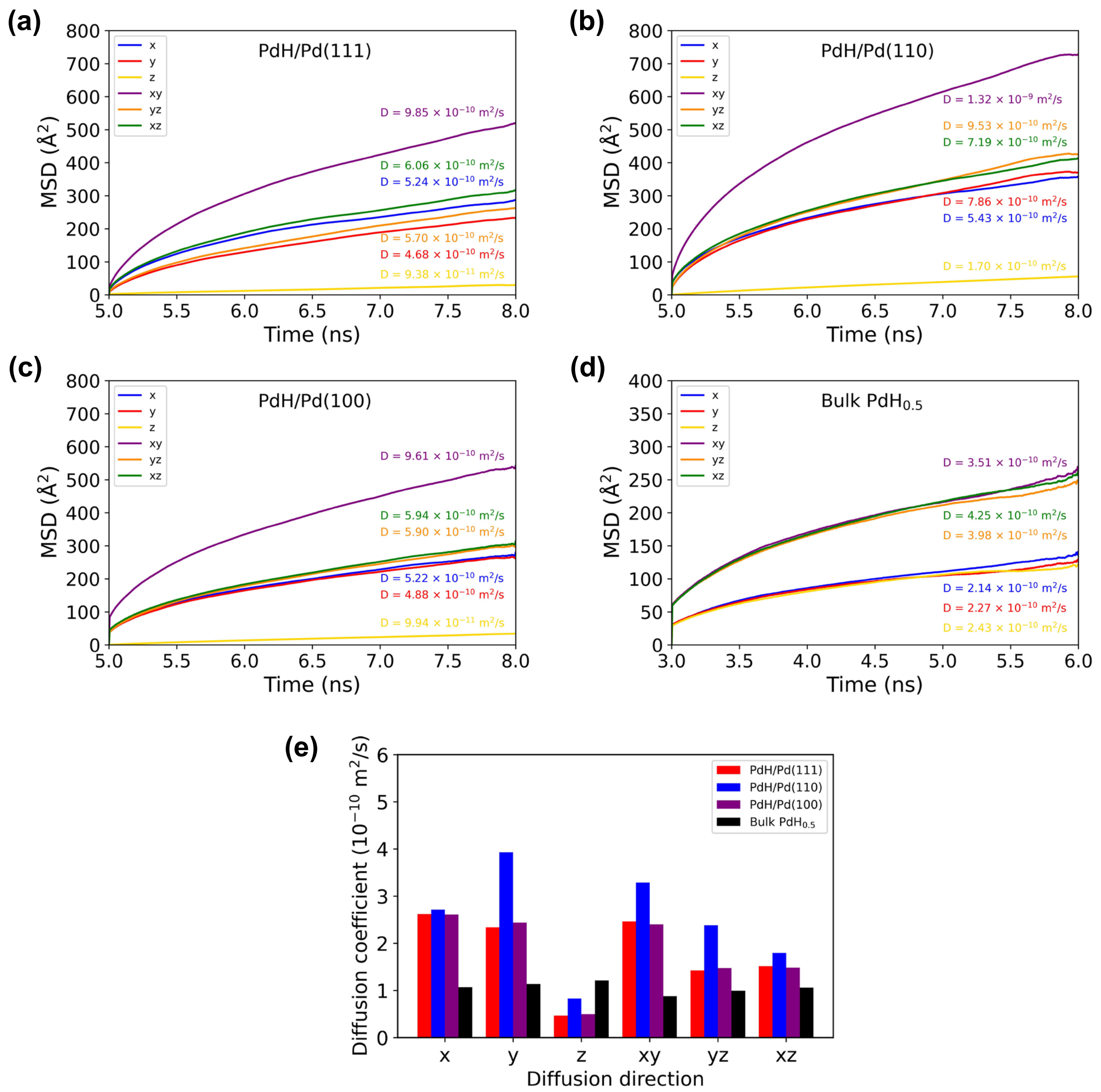

Figure S14. Mean square displacements (MSD) of H during the last 3 ns of MD simulations with the ACE-PBE-D3 potential for a) PdH/Pd(111), b) PdH/Pd(110), c) PdH/Pd(100), and d) bulk $PdH_{0.5}$ systems; e) shows the comparison of the average diffusion coefficient of hydrogens in these systems, where the diffusion coefficient was calculated by fitting the MSD data with respect to the lag-time to a linear model (obtaining R^2 in the range of 0.9924-0.9999).

As illustrated in **Figure S14**, we observed anisotropic diffusion characteristics across the three PdH/Pd(111), PdH/Pd(110), and PdH/Pd(100) systems. For all the surfaces studied, the MSD in *xy* plane is higher than in other planes or directions, indicating greater lateral mobility of H atoms. The smallest MSD values are observed in the *z* direction, reflecting restricted vertical movement. Notably, H atoms on the PdH/Pd(110) surface exhibit the highest MSD and diffusion coefficient in all directions and planes compared to other surfaces, suggesting that this specific surface orientation facilitates the greatest lateral diffusion, perhaps because all atoms were treated as unconstrained, maintaining the lattice constants of Pd in *x* and *y* direction, with only expansion possibilities in the *z*-direction, orthogonal to the *xy*-plane. This anisotropy can also be attributed to the structural and dynamic properties of the different Pd surfaces. The Pd(110) surface has a unique atomic arrangement characterized by rows of atoms with relatively larger inter-row spacing. This configuration facilitates easier movement of H atoms in the *x* and *y* directions and *xy* plane, reducing resistance to diffusion.

The results from the diffusion study align well with the findings on H distribution (**Figure 5**f-h). The relatively facile movement of H atoms across the PdH/Pd(110) surface allows them to disperse among the various Pd(110) layers (see **Figure 5**g). This diffusion behaviour can be attributed to the unique surface orientation of Pd(110), as previously discussed. However, H distribution in Pd(111) indicates a concentration of H atoms in the top-half of the slab (within the 50–80 Å region, layers 10 to 22), where H atoms tend to remain in their initial regions. This localized distribution can be explained by the densely packed structure of Pd(111). The diffusion study further reveals that the diffusion coefficient of H atoms in Pd(111) is lower than in Pd(110) (see **Figure S14**), likely due to the compact arrangement of Pd(111) atoms, corroborating the observed H distribution pattern. Nevertheless, some H atoms in Pd(111) appear to diffuse to the substrate's lower surface, while the bottom-half of the slab (the region between z = 30 to 50 Å) shows minimal H presence. It is suggested that H atoms reoriented Pd(111) layers in the top-half during the simulation, where they were almost initially located. Due to the dense packing in this region, the H atoms caused an expansion (see **Figure 6**a), resulting in a dislocation around layers 10-11 (see **Figure 6**g). However, as shown in **Figure 6**a, the structure in the bottom-half region remains unstrained, indicating limited H diffusion. This suggests that the few H atoms capable of diffusing preferred the accessible bottom surface of Pd(111) over the tightly constrained structure within the bottom-half of the slab. Despite anisotropic diffusion in the Pd(100) slab, hydrogen achieves a uniform distribution (see **Figure 5**h), leading to homogeneous z-axis expansion. This allows strain relief through minor surface

rearrangements, without the need for dislocations, due to the slab's simple symmetry and minimal strain gradients.

In the bulk $PdH_{0.5}$ system, the MSD in planar directions and the slightly higher axial diffusivity can be explained by the energy landscapes faced by H atoms. In the crystal lattice, H atoms encounter different energy barriers along planar and axial directions. While H atoms have more freedom of movement in the planes, leading to higher MSD values, they might find pathways with fewer constraints or lower energy barriers in the axial direction, resulting in higher axial diffusivity. Additionally, the similarity in the planar diffusion components and in the axial diffusion components suggests isotropic diffusion behaviour both within the planes and along the individual axes.

Section S8: Visualization of the local structures and dislocations of the equilibrated PdH/Pd(111) and PdH/Pd(110) systems

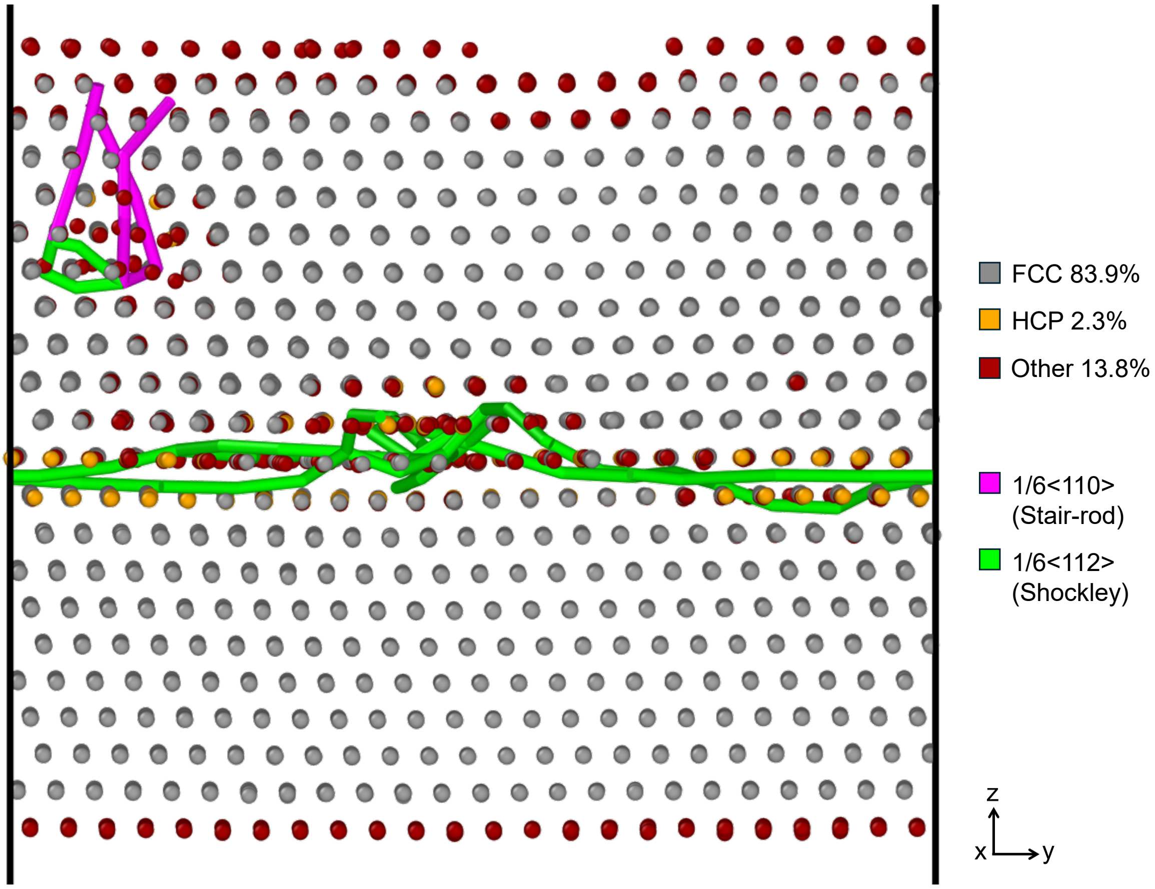

Figure S15. Visualization of the local structure and dislocations of the reoptimized PdH/Pd(111) surface after 8 ns MD equilibration at 300 K using the ACE-PBE-D3 potential. In total 14 dislocation lines are present: the 1/6<112> Shockley partial type occurs more frequently (11 dislocation lines), than the 1/6<110> stair-rod type (3 dislocation lines).

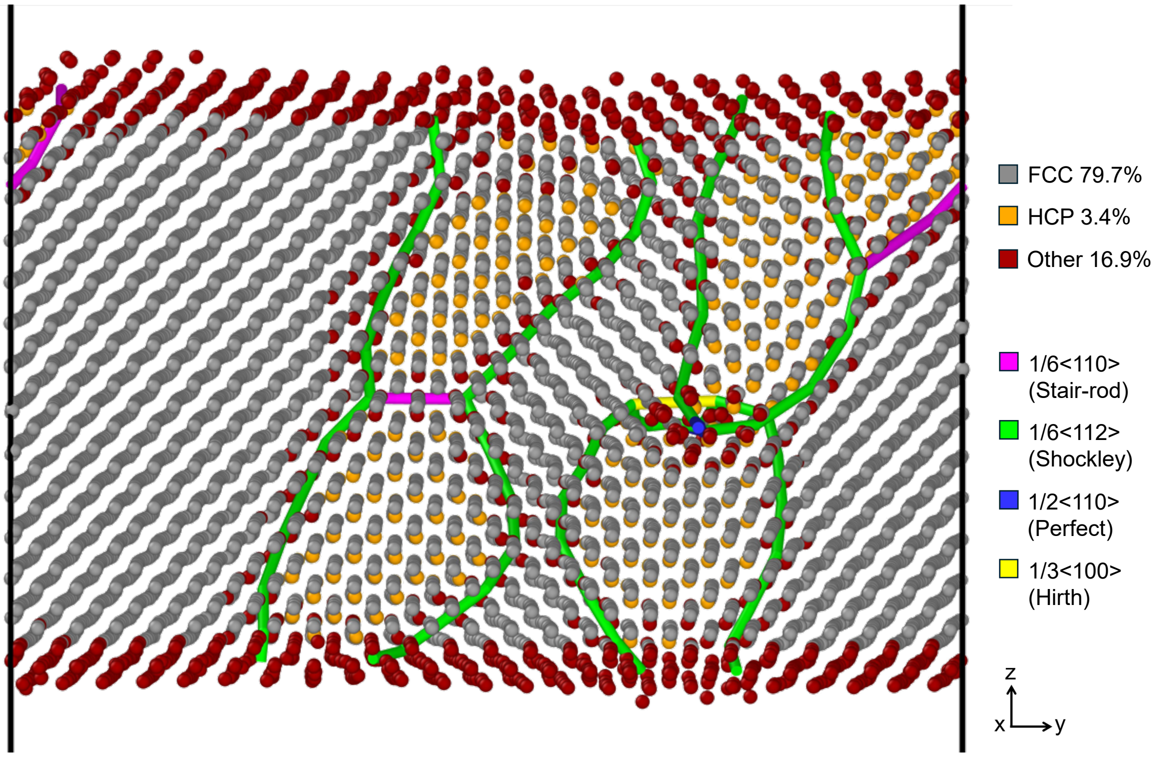

Figure S16. Visualization of the local structure and dislocations of the reoptimized PdH/Pd(110) surface after 8 ns MD equilibration at 300 K using the ACE-PBE-D3 potential. In total 15 dislocation segments are present: the 1/6<112> Shockley partial type (11 dislocation lines) and the 1/6<110> stair-rod type (1 dislocation line) take place along diagonal directions, whereas the 1/2<110> perfect type (1 dislocation line) happens along *x* direction and the 1/6<110> stair-rod type (1 dislocation line) and the 1/3<100> Hirth type (1 dislocation line) exist along *y* direction.

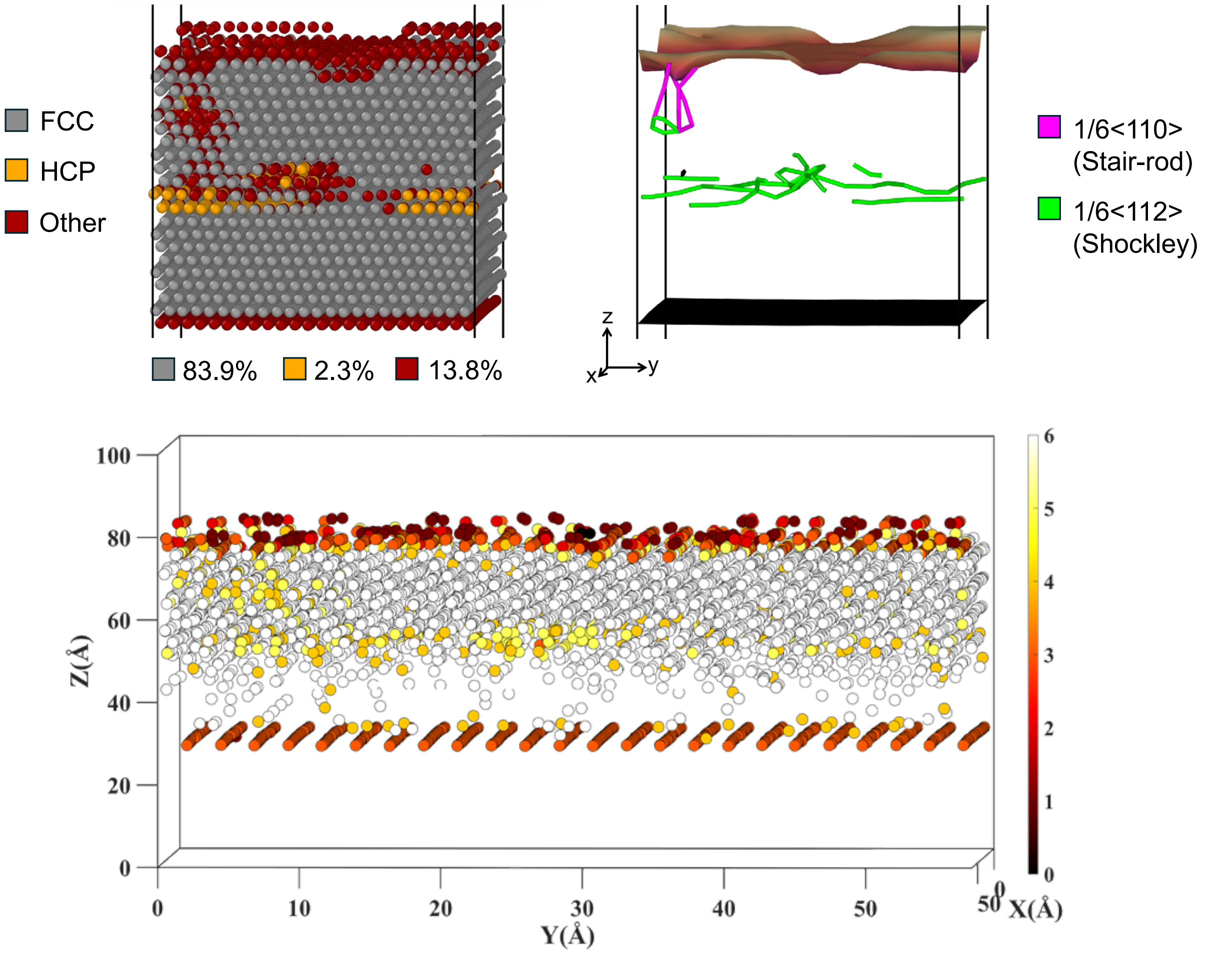

Figure S17. Visualization of the local structure and dislocations of the PdH/Pd(111) surface after 8 ns MD equilibration at 300 K using the ACE-PBE-D3 potential (upper panel); and the H atoms colored by their Pd nearest neighbors (lower panel), see details of this analysis in **Section S5**.

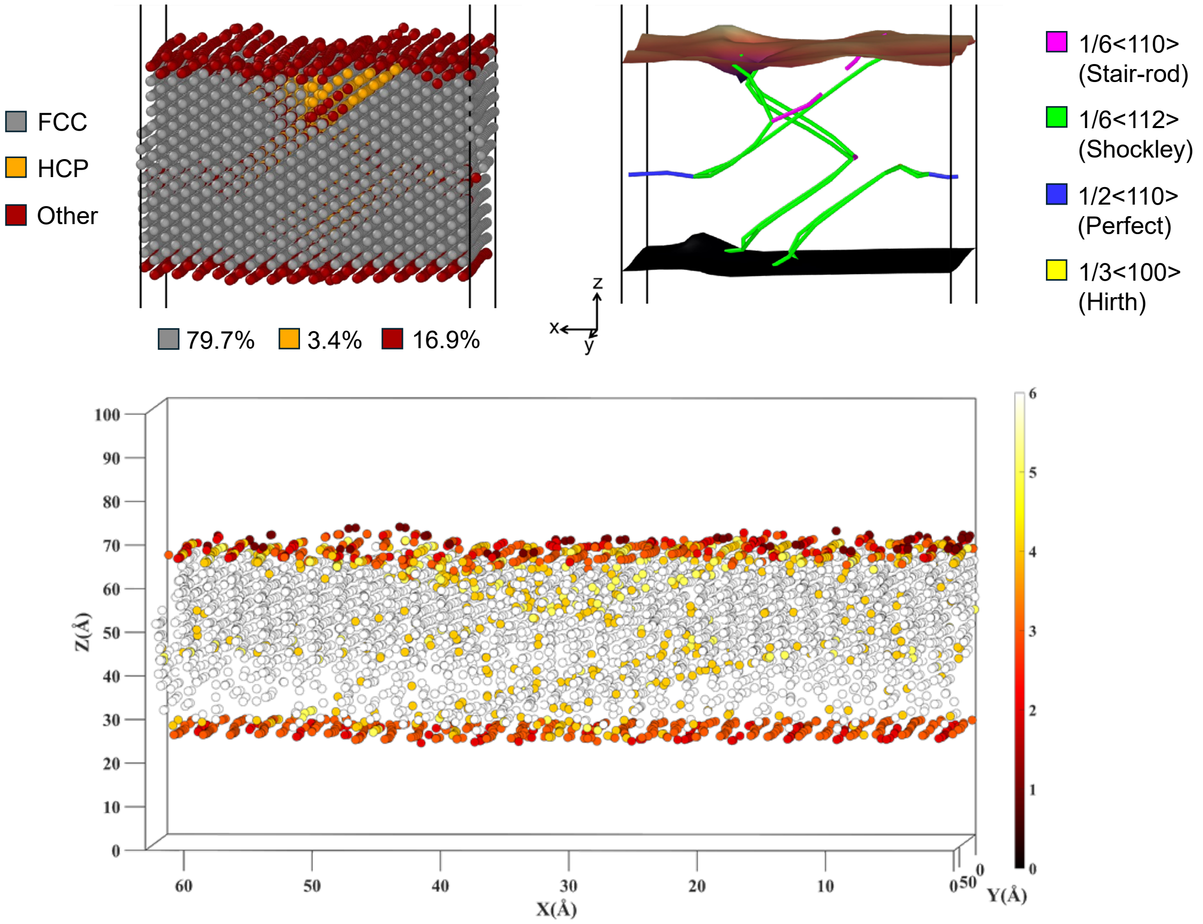

Figure S18. Visualization of the local structure and dislocations of the PdH/Pd(110) surface after 8 ns MD equilibration at 300 K using the ACE-PBE-D3 potential (upper panel); and the H atoms colored by their Pd nearest neighbors (lower panel), see details of this analysis in **Section S5**.

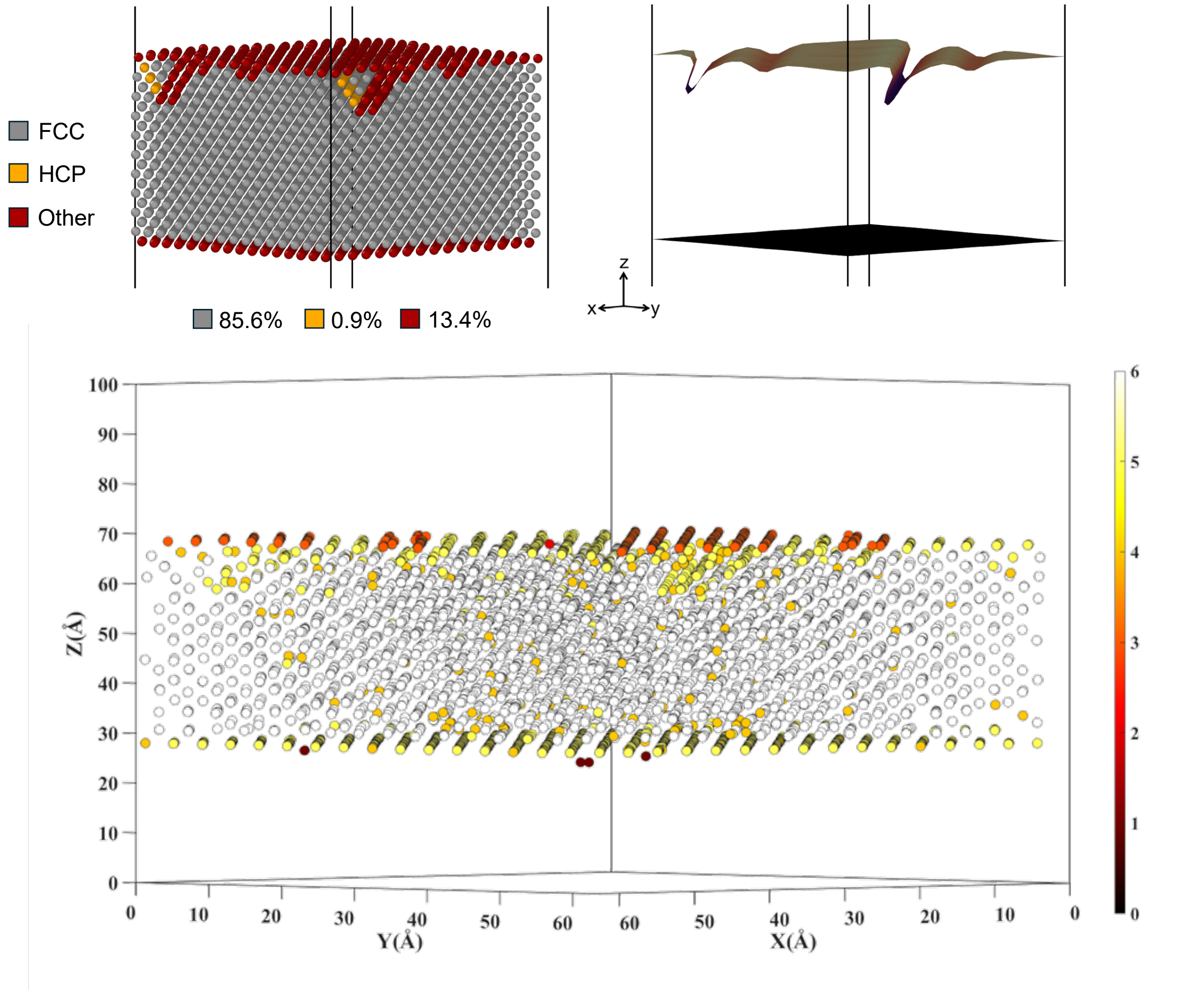

Figure S19. Visualization of the local structure and dislocation of the PdH/Pd(100) surface after 8 ns MD equilibration at 300 K using the ACE-PBE-D3 potential (upper panel); and the H atoms colored by their Pd nearest neighbors (lower panel), see details of this analysis in **Section S5**.

Section S9: Characterization of the Pd NPs supported on Vulcan XC-72

The metal content of Pd NPs supported on Vulcan XC72 was determined by ICP-MS. The measure was performed on a PerkinElmer NexION 2000. Approximately 3 mg of material was precisely weighted and dissolved using microwave-assisted digestion (Mars 6, CEM corporation, EasyPrep Plus vessels) in 10 mL aqua regia. The Pd fraction measured in the commercial and home-made samples was determined to be 17.5 wt.% and 20.1 wt.% respectively.

The TEM images were acquired, respectively, using a JEOL 2010 (LaB_6 filament with a resolution of 0.19 nm) operated at 200 kV. The number-averaged mean particle size was obtained through statistical analysis by manually counting isolated Pd nanoparticles using the ImageJ software. The mean particle diameter of the commercial 4nm-Pd/C sample was determined to be 3.6 ± 0.7 nm from the statistical analysis of 500 particles (see **Figure S20**a), while the one of the home-made 14nm-Pd/C sample was measured at 14.1 ± 2.4 nm from the statistical analysis of 120 particles (see **Figure S20**b).

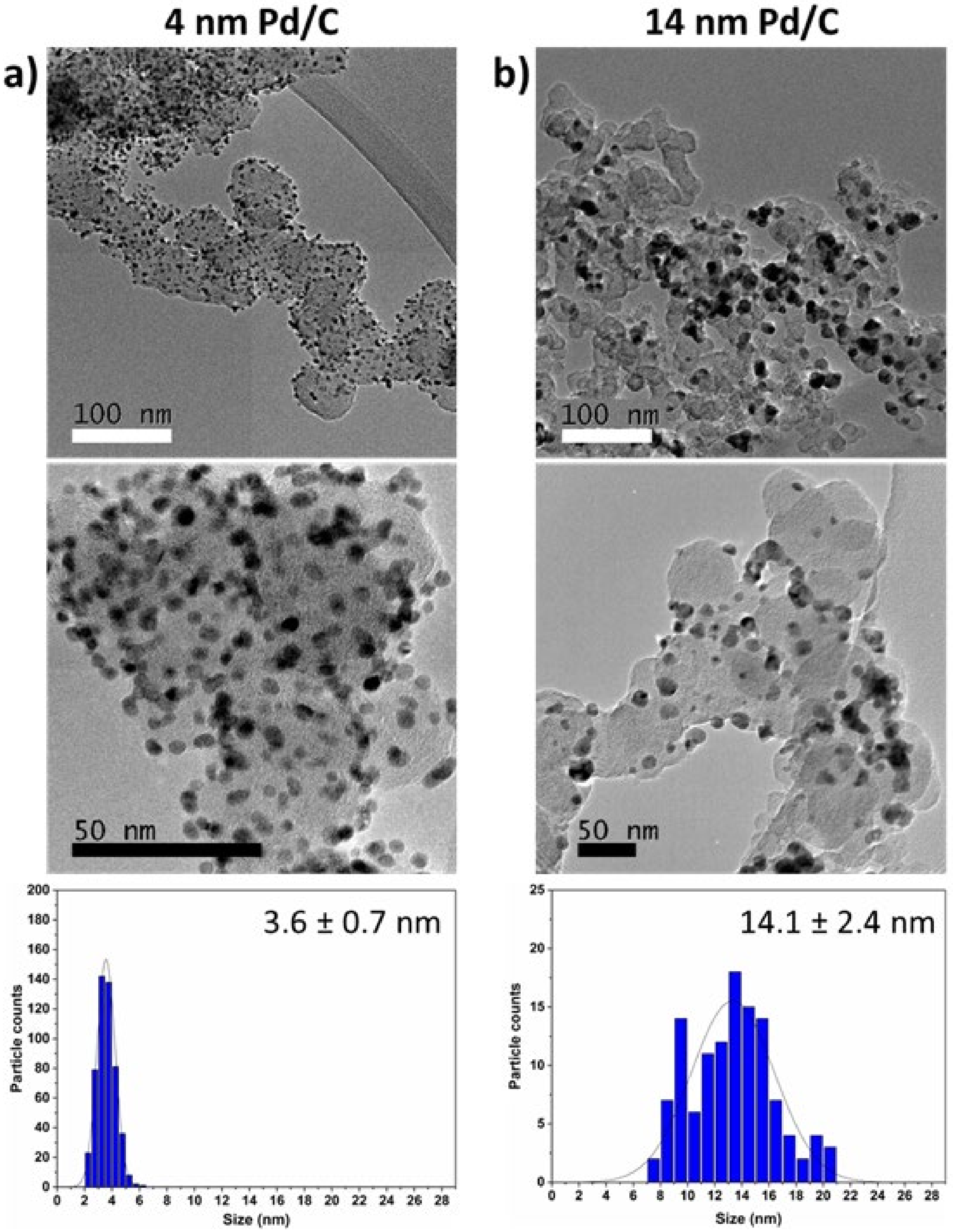

Figure S20. Typical TEM images (white scale bar = 100 nm, black scale bar = 50 nm) and corresponding particle size distributions (blue bars: raw data, line: fitted normal distribution) of a) the 4 nm commercial, and b) 14 nm home-made catalysts.

Section S10: Validation of the atomic cluster expansion (ACE) potential

The ACE potential is validated thoroughly to ensure that it reproduces the fundamental properties of Pd and PdH crystals. Key properties of interest such as elastic properties, vibrational properties, surfaces and hydrogen diffusion and segregation are predominantly represented by structures having low formation energies. Therefore, all structures within 2 eV/atom above the ground state are given 95% higher relative weights. High-energy structures, meanwhile, only contribute to the model's transferability and enable it to effectively capture repulsive interactions at short interatomic distances.

The final ACE potential achieves an energy RMSE of 9 meV/atom for low-energy structures and 39 meV/atom across the complete training dataset. Corresponding force RMSE values are 65 meV/Å for low-energy structures and 97 meV/Å for the full dataset. A split-test set, representing 10% of the reference data, is excluded from training and reserved for validation. **Figure S21**a,b show the cross-validation of the predicted energies and forces, respectively, over the split-test-set, along with the error distributions. A dotted line in **Figure S21**a indicates the energy cutoff for low-energy structures, highlighting that all predicted energies below this threshold align well with reference data. While a small subset of high-energy points exhibits some deviation, these represent, high-energy structures included primarily to approximate the potential's repulsive behavior. Consequently, limited accuracy on these points does not impact the reliability of key properties.

Figure S21c illustrates the near-equilibrium energy-volume curves for pure Pd, $PdH_{0.5}$, and PdH_1 crystals, as predicted by ACE together with the reference DFT calculations, showing excellent agreement. **Figure S21**d shows the predicted bulk modulus and equilibrium lattice constant for fcc-Pd, as predicted by ACE, DFT and experimentally reported values.[7-8] The close agreement with experimental data guided the choice of a PBE exchange-correlation functional with the D3 dispersion in reference DFT calculations. **Figure S21**e,f show the phonon band structure of fcc-Pd and stoichiometric PdH, respectively. While minor deviations are noted for PdH, the ACE-predicted phonon band structure for fcc-Pd shows excellent agreement with the reference DFT results.

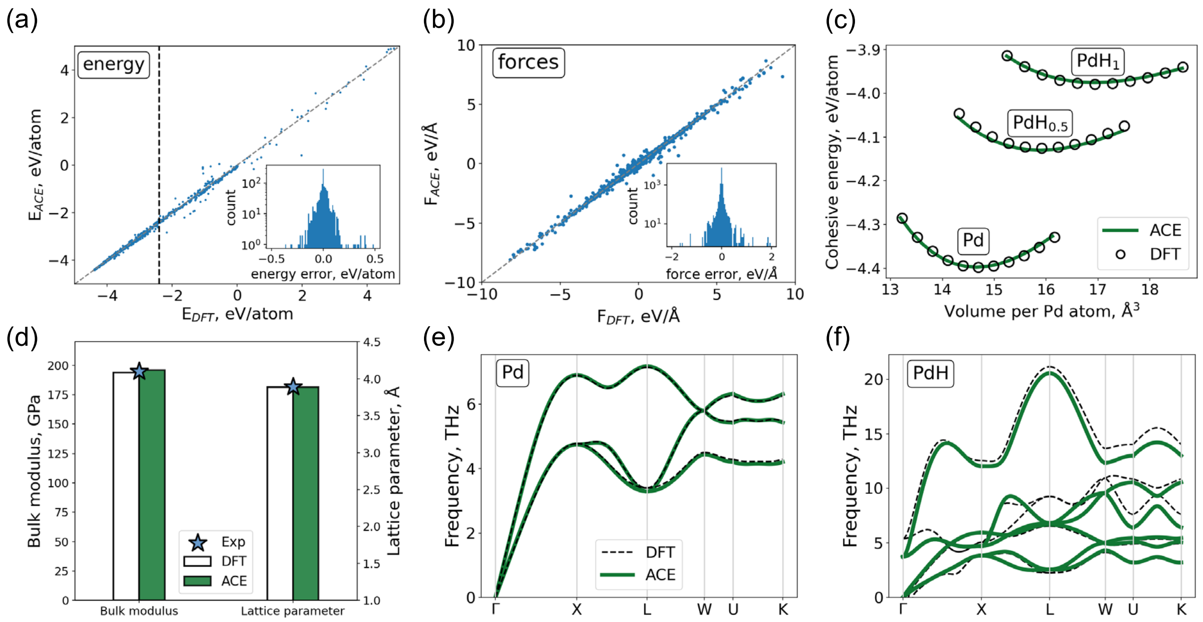

Figure S21. Validation of the ACE potential; a) Cross-validation plots and associated error distributions for the predicted energies, with the vertical line indicating the energy cutoff below which all structures receive high relative weights during training, b) Cross-validation plots and error distributions for predicted forces, c) Energy-volume curves calculated by ACE, compared with reference DFT for Pd crystals with varying hydrogen concentrations (Pd, $PdH_{0.5}$, and PdH_1), d) Comparison of bulk modulus and equilibrium lattice constants predicted by ACE and DFT with experimentally reported values,[7-8] e) Phonon band structure along high-symmetry points of the Brillouin zone for fcc-Pd and f) stoichiometric PdH, demonstrating close agreement between ACE predictions and DFT, thereby capturing essential vibrational properties.

Section S11: MD simulations of the PdH/Pd(hkl) systems using the ACE-PBE potential

MD simulations based on the ACE-PBE potential were performed on the PdH/Pd(111), PdH/Pd(110), and PdH/Pd(100) surfaces. The reoptimized structures of these systems after 8 ns MD equilibration at 300 K are illustrated in **Figure S22**a-c. Formation of defects, surface reconstruction, and surface roughening were observed on all three surfaces, as similarly found in results obtained by the ACE-PBE-D3 potential (see **Figure 5**a-c). **Figure S22**d shows that the roughening of all three surfaces appears already during the first ns of MD simulations, where the roughness values of ~1.07 Å, ~0.89 Å, and ~0.78 Å were found at 8 ns for the Pd(111), Pd(110), and Pd(100) surfaces, respectively. A similar trend is obtained with the ACE-PBE potential, [(100), ~0.27 Å < (110) ~0.94 Å < (111) ~1.53 Å]. However, for the equilibrated

PdH/Pd(100) system, the surface is more distorted with the creation of vacancies on the surface (**Figure S22**c) compared to the ACE-PBE-D3 treated systems (see **Figure 5**c), resulting in larger roughness (~0.78 Å, ACE-PBE vs ~0.27 Å, ACE-PBE-D3).

Notably, ACE-PBE and ACE-PBE-D3 potentials provide different H absorption behaviour, where H atoms occupy more tetrahedral interstitial sites in case of ACE-PBE treated surfaces instead of occupying mostly the octahedral sites as found from the ACE-PBE-D3 simulations (compare **Figure S22**e and **S23** for the ACE-PBE results with **Figure 5**e and **Figures S17-S19** for the ACE-PBE-D3 results). From the distributions of Pd atoms along *z* direction as shown in **Figure S22**f-h (details of this analysis are provided in **Section S6**), the number of Pd atoms varies for all studied systems, implying that some subsurface Pd atoms migrate to the surface which leads to the surface reconstruction and surface roughening. Furthermore, as similarly found in the ACE-PBE-D3 results, H diffuses into deeper subsurface layers, eventually populating another surface side (**Figure S22**f-h) and H content is higher on the surfaces than on the subsurface layers for all systems (see **Figure S24**). The H diffusion in these systems were also investigated by calculating the MSD and diffusion coefficient as presented in **Figure S25**. Details of these calculations are provided in **Section S7**. **Figure S25**e shows that H diffuses fastest in *xy* direction for all surface systems. The diffusion coefficient of H in different directions in all systems obtained between ACE-PBE-D3 and ACE-PBE potentials were compared as shown in **Figure S26**. In comparing the planar diffusion coefficients (D_{xy}, D_{yx}, and D_{xz}) between the system simulated with the ACE-PBE-D3 potential and those simulated with PBE, we observed that the former exhibits lower values. However, in the bulk $PdH_{0.5}$, the simulated systems with both potentials exhibit nearly identical planar diffusion coefficients. Furthermore, the analysis of strains, structural changes, and dislocations for the studied surface systems simulated by the ACE-PBE potential provides similar results as found for the ACE-PBE-D3 ones, except for the (111) system (compare **Figure S27** and **Figure 6**). Unlike the ACE-PBE-D3 (100) system, the equilibrated PdH/Pd(100) obtained by ACE-PBE potential exhibits dislocations in the upper-half sublayers (see **Figure S27**i). **Table S3** summarizes the fraction of phase transformations and dislocation occurring in structures obtained by two different potentials.

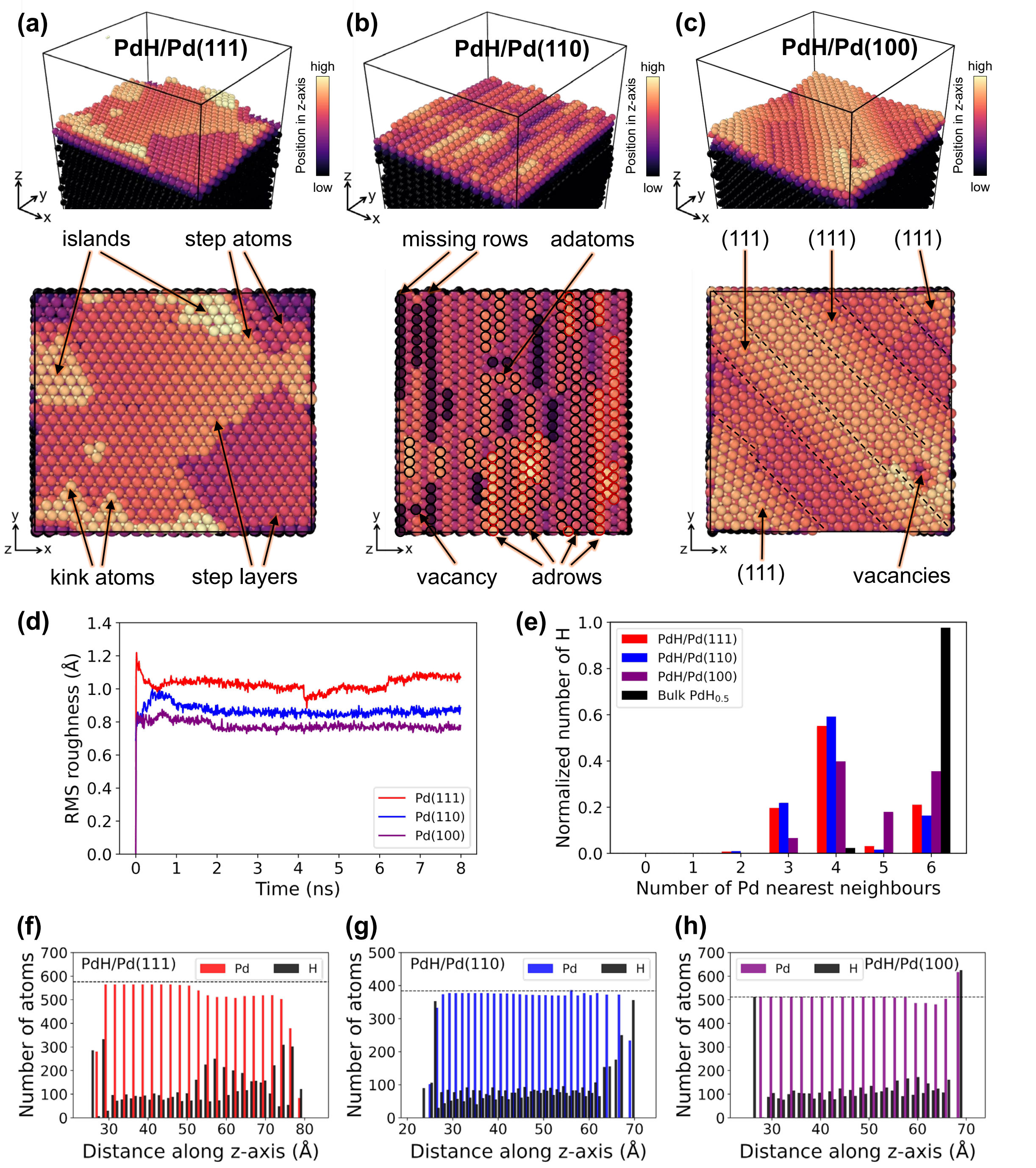

Figure S22. Surface analysis of the reoptimized structures after 8 ns MD at 300 K using the ACE-PBE potential with color gradient indicating the atomic position along the *z* direction for a) PdH/Pd(111), b) PdH/Pd(110), and c) PdH/Pd(100), d) Calculated root mean square (RMS) roughness of the three Pd surfaces as a function of simulation time, e) Number of H atoms surrounded by different number of Pd nearest neighbors for the three reoptimized structures after 8 ns MD, which is normalized by the total number of H in each system. The nearest neighbor analysis for bulk $PdH_{0.5}$ is also presented for comparison, f-h) Distributions of Pd and H atoms along *z* direction in these model systems, where dashed lines indicate the number of Pd per layer in the initial structures.

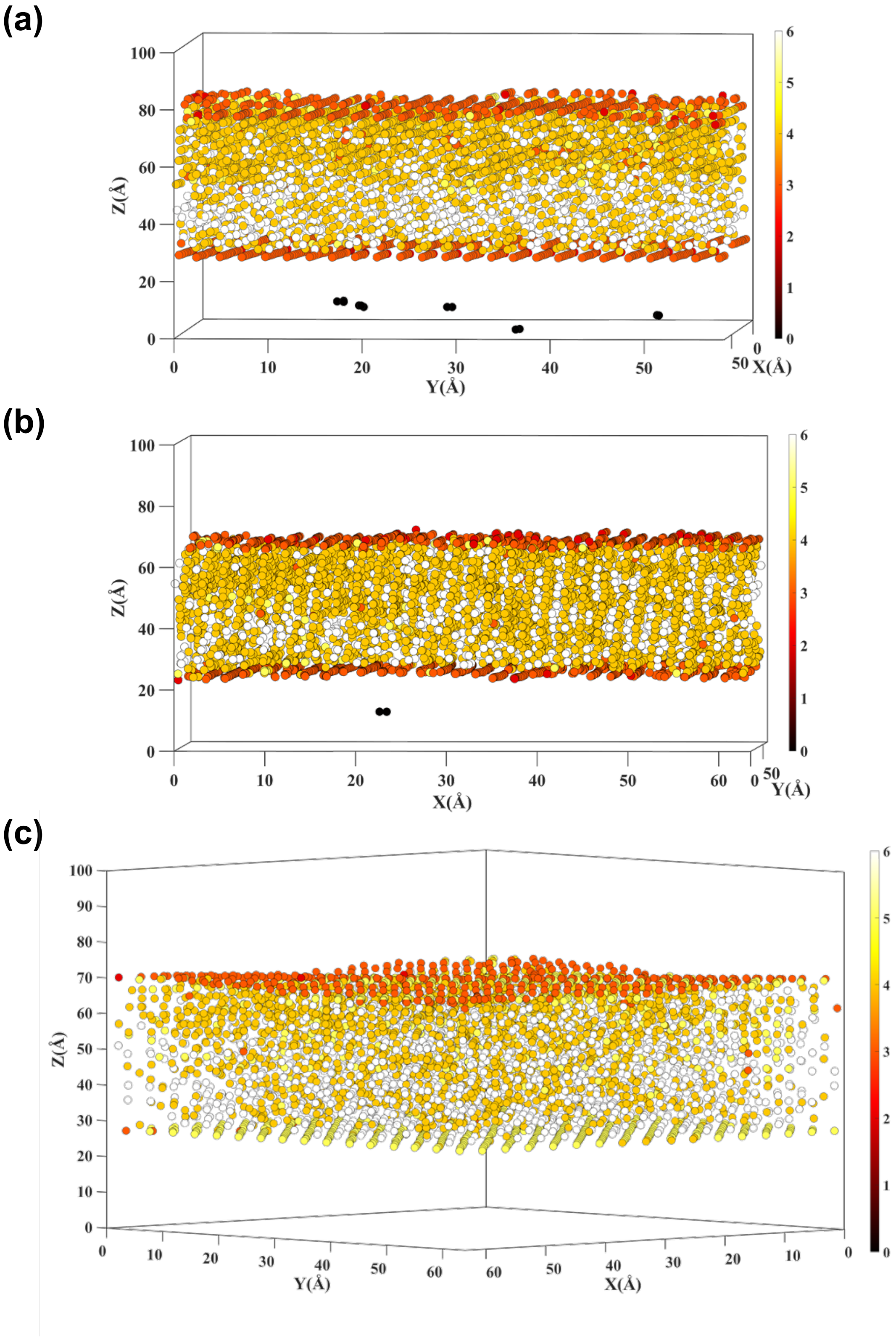

Figure S23. The H atoms colored by their Pd nearest neighbors of the reoptimized structures after 8 ns MD at 300 K using the ACE-PBE potential for a) PdH/Pd(111), b) PdH/Pd(110), and c) PdH/Pd(100), see details of this analysis in **Section S5**.

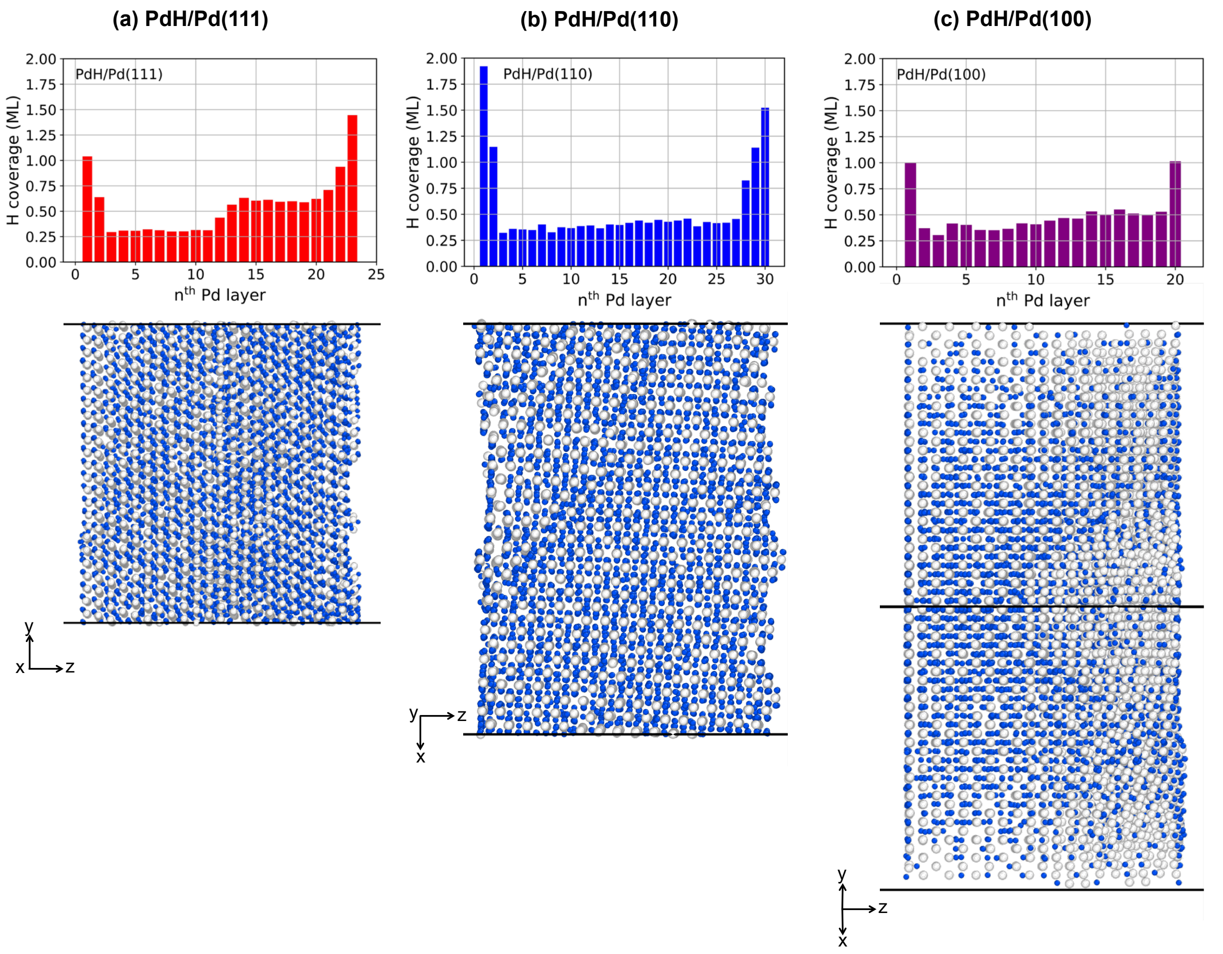

Figure S24. H content (in units of monolayer (ML)) on each Pd layer in the reoptimized structures after 8 ns MD equilibration at 300 K using the ACE-PBE potential for a) PdH/Pd(111), b) PdH/Pd(110), and c) PdH/Pd(100), see details of this analysis in **Section S6**. Visualization of these model systems in *z* direction is also presented in the lower panel, where the white and blue balls represent Pd and H atoms, respectively.

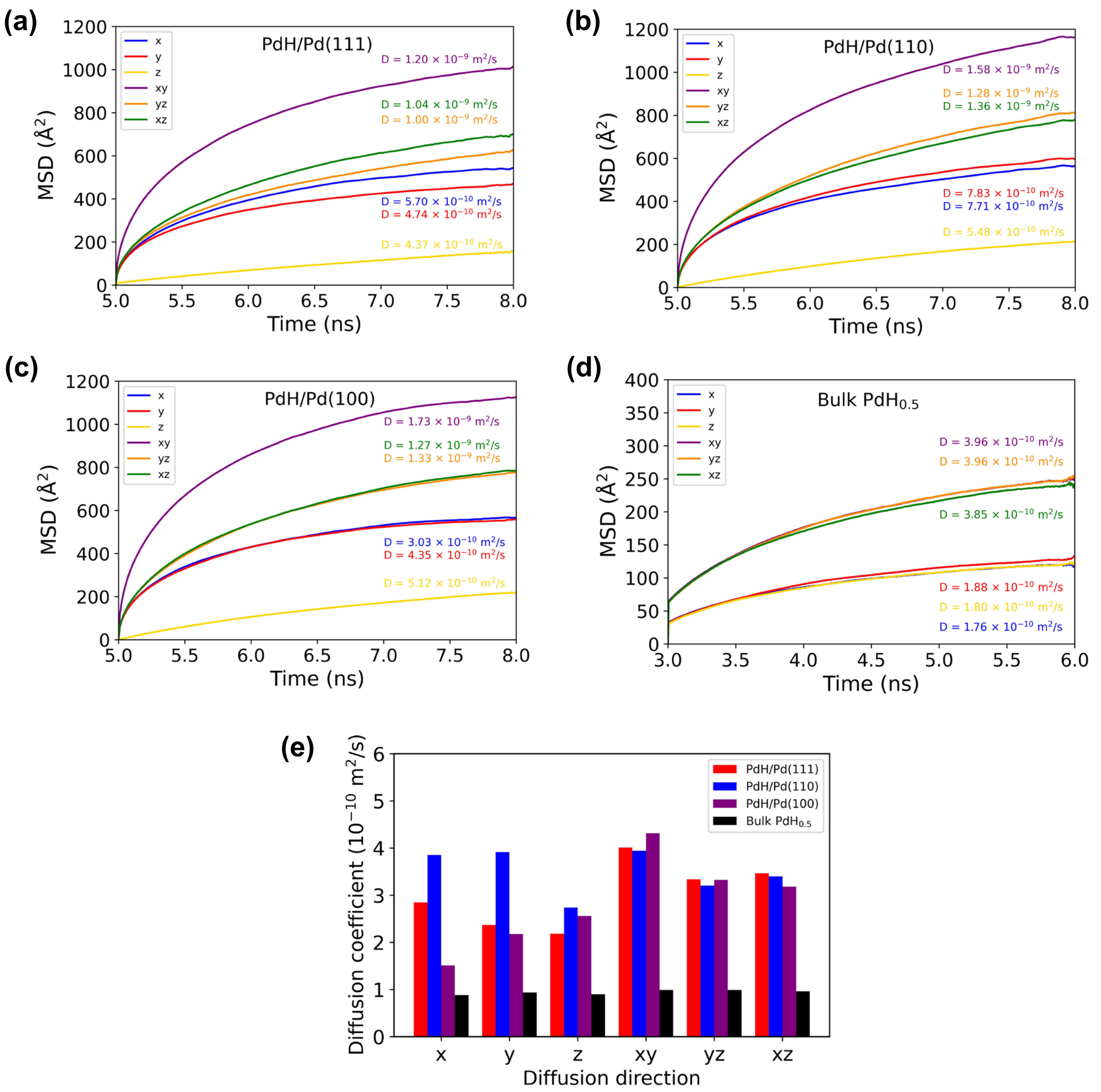

Figure S25. Mean square displacements (MSD) of H during the last 3 ns of MD simulations using the ACE-PBE potential of a) PdH/Pd(111), b) PdH/Pd(110), c) PdH/Pd(100), and d) bulk $PdH_{0.5}$ systems; e) shows the comparison of the hydrogen diffusion coefficient between these systems, where the diffusion coefficient was calculated by fitting the MSD data with respect to the lag-time to a linear model (obtaining R^2 in the range of 0.9807-0.9989), see details of this analysis in **Section S7**.

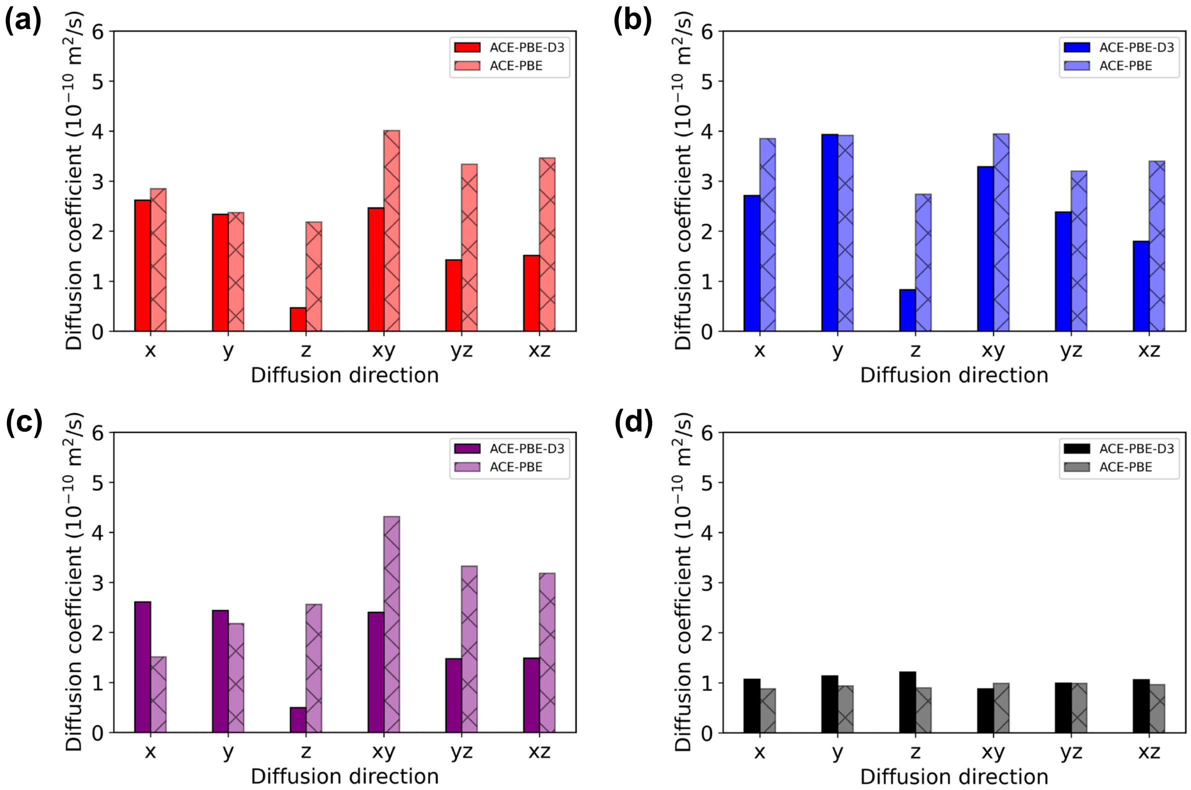

Figure S26. Comparison of the hydrogen diffusion coefficient in different directions obtained from simulations with the ACE-PBE-D3 and ACE-PBE potentials for a) PdH/Pd(111), b) PdH/Pd(110), c) PdH/Pd(100), and d) bulk $PdH_{0.5}$ systems.

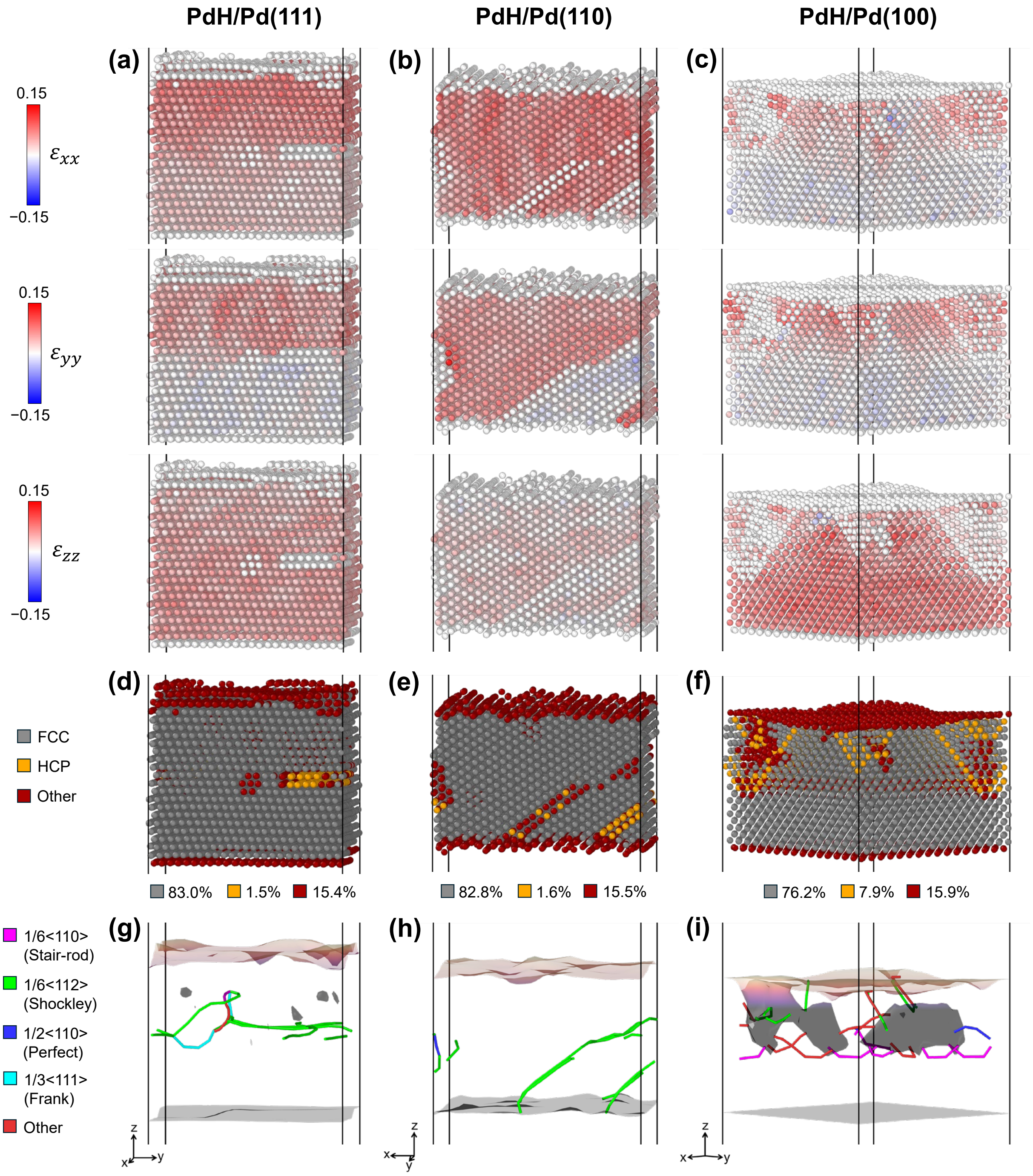

Figure S27. The elastic strain (ε) for each Pd atom along x-, y-, and z-axis (H atoms omitted for clarity) of the reoptimized structures after 8 ns MD at 300 K using the ACE-PBE potential for a) PdH/Pd(111), b) PdH/Pd(110), and c) PdH/Pd(100), evaluated by determining the total deformation of the crystal around each atom, with respect to an fcc Pd with a lattice constant of 3.891 Å, where positive and negative values indicate the tensile and compressive strains, respectively; d-f) visualization of the local structures and g-i) dislocations of these model systems.

Table S3. Comparison of results on structural and dislocation analysis of the reoptimized structures after 8 ns MD equilibration at 300 K for the PdH/Pd(111), PdH/Pd(110), and PdH/Pd(100) surfaces, using the ACE-PBE-D3 and ACE-PBE potentials.

**Analysis**	**ACE-PBE-D3 potential**			**ACE-PBE potential**		
	**PdH/Pd(111)**	**PdH/Pd(110)**	**PdH/Pd(100)**	**PdH/Pd(111)**	**PdH/Pd(110)**	**PdH/Pd(100)**
**Structural fraction (%)**						
FCC	83.9	79.7	85.6	83.0	82.8	76.2
HCP	2.3	3.4	0.9	1.5	1.6	7.9
Other	13.8	16.9	13.4	15.4	15.5	15.9
**Dislocation type (segments [length])**						
1/6<110> (Stair-rod)	3 [38.731]	2 [31.433]	0	1 [11.104]	0	5 [82.435]
1/6<112> (Shockley)	11 [261.793]	11 [286.793]	0	12 [245.224]	8 [181.203]	7 [90.754]
1/2<110> (Perfect)	0	1 [18.569]	0	0	1 [6.688]	1 [14.244]
1/3<100> (Hirth)	0	1 [5.871]	0	0	0	0
1/3<111> (Frank)	0	0	0	2 [27.1189]	0	0
Other	0	0	0	2 [11.933]	0	10 [114.274]
Total	14 [300.524]	15 [342.666]	0	17 [295.38]	9 [187.892]	23 [301.708]

References

[1] E. S. Gadelmawla, M. M. Koura, T. M. A. Maksoud, I. M. Elewa, H. H. Soliman, *J. Mater. Process. Technol.* **2002**, 123, 133.

[2] A. Viola, R. Chattot, V. Martin, G. Tsirlina, J. Nelayah, J. Drnec, F. Maillard, *J. Phys. Chem. C* **2023**, 127, 17761.

[3] A. Ngoipala, R. Lipin, R. L. Arevalo, M. Vandichel, *Int. J. Hydrogen Energy.* **2024**, 53, 829.

[4] S. Lloyd, *IEEE Trans. Inf. Theory* **1982**, 28, 129.

[5] L. R. Evangelista, E. K. Lenzi, G. Barbero, A. M. Scarfone, *Phys. A: Stat. Mech. Appl.* **2024**, 635, 129491.

[6] M. A. Islam, *Phys. Scr.* **2004**, 70, 120.

[7] N. V. Ilawe, J. A. Zimmerman, B. M. Wong, *J. Chem. Theory Comput.* **2015**, 11, 5426.

[8] G. I. Csonka, J. P. Perdew, A. Ruzsinszky, P. H. T. Philipsen, S. Lebègue, J. Paier, O. A. Vydrov, J. G. Ángyán, *Phys. Rev. B* **2009**, 79, 155107.
